# A comparative study on ctDNA and tumor DNA mutations in lung cancer and benign cases with a high number of CTCs and CTECs

**DOI:** 10.1186/s12967-023-04746-8

**Published:** 2023-12-01

**Authors:** Jianzhu Xie, Binjie Hu, Yanping Gong, Sijia He, Jun Lin, Qian Huang, Jin Cheng

**Affiliations:** 1 Cancer Center, Shanghai General Hospital, Shanghai Jiao Tong University School of Medicine, Shanghai, China; 2 Department of Pathology, Shanghai General Hospital, Shanghai Jiao Tong University School of Medicine, Shanghai, China

**Keywords:** Lung cancer, ctDNA, Mutations, NGS, CTCs, CTECs

## Abstract

**Background:**

Liquid biopsy provides a non-invasive approach that enables detecting circulating tumor DNA (ctDNA) and circulating tumor cells (CTCs) using blood specimens and theoretically benefits early finding primary tumor or monitoring treatment response as well as tumor recurrence. Despite many studies on these novel biomarkers, their clinical relevance remains controversial. This study aims to investigate the correlation between ctDNA, CTCs, and circulating tumor-derived endothelial cells (CTECs)  while also evaluating whether mutation profiling in ctDNA is consistent with that in tumor tissue from lung cancer patients. These findings will help the evaluation and utilization of these approaches in clinical practice.

**Methods:**

104 participants (49 with lung cancer and 31 with benign lesions) underwent CTCs and CTECs detection using integrating subtraction enrichment and immunostaining-fluorescence in situ hybridization (SE-iFISH) strategy. The circulating cell-free DNA (cfDNA) concentration was measured and the mutational profiles of ctDNA were examined by Roche AVENIO ctDNA Expanded Kit (targeted total of 77 genes) by next generation sequencing (NGS) in 28 patients (20 with lung cancer and 8 with benign lesions) with highest numbers of CTCs and CTECs. Mutation validation in matched tumor tissue DNA was then performed in 9 patients with ctDNA mutations using a customized xGen pan-solid tumor kit (targeted total of 474 genes) by NGS.

**Results:**

The sensitivity and specificity of total number of CTCs and CTECs for the diagnosis of NSCLC were 67.3% and 77.6% [AUC (95%CI): 0.815 (0.722–0.907)], 83.9% and 77.4% [AUC (95%CI): 0.739 (0.618–0.860)]. The concentration of cfDNA in plasma was statistically correlated with the size of the primary tumor (r = 0.430, *P* = 0.022) and CYFRA 21–1 (r = 0.411, *P *= 0.041), but not with the numbers of CTCs and CTECs. In this study, mutations were found to be poorly consistent between ctDNA and tumor DNA (tDNA) in patients, even when numerous CTCs and CTECs were present.

**Conclusion:**

Detection of CTCs and CTECs could be the potential adjunct tool for the early finding of lung cancer. The cfDNA levels are associated with the tumor burden, rather than the CTCs or CTECs counts. Moreover, the poorly consistent mutations between ctDNA and tDNA require further exploration.

**Supplementary Information:**

The online version contains supplementary material available at 10.1186/s12967-023-04746-8.

## Background

Lung cancer ranks second as one of the most common malignant tumors worldwide, with an incidence rate of 31.5 per 100,000 in males and 14.6 per 100,000 in females [1]. In China, the mortality rate is significantly higher than that in the United States (about 1.4 times), even though the incidences are similar [2]. Furthermore, the five-year survival rate is relatively lower, compared with developed countries, which may be caused by the lack of national-scale lung cancer screening and insufficient population coverage [2]. In this regard, effective screening and early diagnosis have increasingly become the keys to reducing the mortality of lung cancer.

Tissue biopsy remains the gold standard but can be restricted by implementation conditions such as unavailable or inadequate tumor tissues and poor compliance [3]. Currently, low-dose computed tomography (LDCT) scanner is the primary screening method for early-stage lung cancer. Numerous large-scale clinical trials have demonstrated a significant reduction in mortality rates accompanying routine LDCT screening [4, 5]. However, LDCT is limited by the high false positive rate and unnecessary radiation exposure and there is still a need for further confirmation by pathological diagnosis. Consequently, LDCT is not utilized as a standalone method for lung caner screening in the majority of European countries [6]. Therefore, non-invasive and effective tools for lung cancer diagnosis are urgently needed.

Multiple biomarkers in the peripheral blood such as shed tumor cells and cancer-derived molecules have been explored by scientists in order to provide more valuable information, known as liquid biopsy [6–9]. Among these, circulating tumor cells (CTCs) have sparked considerable interest as a potential biomarker for monitoring cancer presence and progression. Furthermore, a subset of these cells has undergone epithelial-mesenchymal transition (EMT) or other phenotype alterations to evade immune surveillance, potentially contributing to distant metastasis [10, 11]. Detection of CTCs is still challenging due to their low numbers in the bloodstream, which makes it difficult to distinguish them from other cells. Despite these obstacles, researchers have made significant progress in developing new technologies and methods for the isolation and identification of CTCs. One promising development is the creation of microfluidic devices designed to quickly isolate CTCs from blood samples [12, 13]. Other progress involve capturing and recognizing CTCs as well as circulating tumor-derived endothelial cells (CTECs) using tumor cell-specific antibodies and probes, and identifying tumor-related mutated gene or various genetic modifications such as methylation through sequencing or PCR [14–19].

Circulating tumor DNA (ctDNA), which is defined as extracellular DNA molecules either passive released into body fluids by apoptotic, necrotic, pyroptotic tumor cells or active released by tumor cells [20], can be a promising tumor biomarker. The molecular diagnostic test of key tumor gene mutations as well as drug-targeted mutations in patients who failed tissue test has been widely adopted [21–23]. One of the earliest applications of ctDNA detection is to identify epidermal growth factor receptor (*EGFR*) mutations for the guidance of *EGFR* inhibitor therapy in non-small cell lung cancer (NSCLC), which has been approved by the Food and Drug Administration (FDA) [24] and further included in the new College of American Pathologists (CAP)/International Association for the Study of Lung Cancer (IASLC)/Association for Molecular Pathology (AMP) testing guidelines [25]. Additionally, there is evidence to support that methylation profiles of ctDNA can enhance both diagnostic and monitoring capabilities for lung cancer [26–28]. Other researchers also confirmed that the presence of mutated ctDNA after radiotherapy or chemotherapy can predict the existence of minimal residual lesions, which may be directly related to tumor metastasis and recurrence [29, 30]. Nevertheless, studies on the concordance of variant profiles between peripheral blood ctDNA and tumor DNA (tDNA) remain controversial [21, 31]. Furthermore, several hurdles still need to be overcome in ctDNA testing, including low concentration in asymptomatic patients, biological contamination from white blood cells, and variations in sensitivity across different platforms [32].

Although new non-invasion biomarkers for lung cancer have become a research hotspot, the relationship between these novel biomarkers is still not well understood. In this research, we aim to investigate the feasibility of CTCs and  CTECs for the diagnosis of NSCLC. We will also examine the coincidence of mutational profiles between ctDNA and tDNA in both lung cancer and benign lung lesion patients, with a particular focus on those with a high number of CTCs and CTECs.

## Methods

### Subjects and specimens

**Fig. 1:**
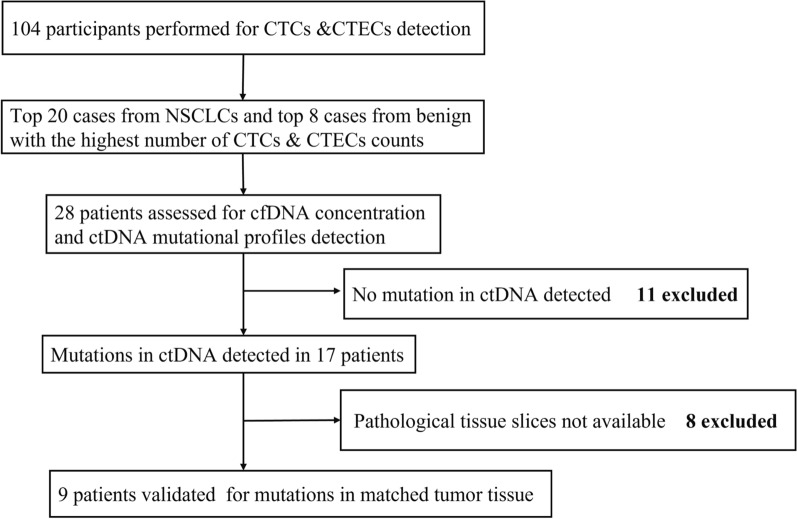
Flow chart detailing the inclusion and exclusion procedures

A total of 104 participants were enrolled in this retrospective study at the Cancer Center in Shanghai General Hospital from August 2017 to April 2019, including 49 cases of newly diagnosed or relapsed non-small cell lung cancer, 31 cases of benign pulmonary disease, and 24 healthy controls. The diagnoses of all the patients were confirmed by clinicians and pathologists. Detailed clinical information is described in Table 1. At first, all the study subjects underwent CTCs and CTECs detection. We further selected the top 20 and 8 patients with the highest number of CTCs and CTECs from NSCLC and benign cases respectively, and ctDNA mutational profiling detection was carried out in these 28 patients. Of these, ctDNA mutations were detected in 17 patients. Among the 17 patients, 9 patients with paired tissue samples underwent tissue DNA sequencing. Specific exclusion criteria are detailed in Fig. 1. In addition, 4 benign patients with ctDNA variants underwent white blood cell targeted sequencing using 32 Gene and 61 Gene panel. The use of all the specimens from patients was in accordance with the Declaration of Helsinki and approved by the Ethical Review Board of Shanghai General Hospital, Shanghai Jiaotong University School of Medicine, China (2016KY130).

**Table 1:** Clinical information of the 104 participating subjects

Characteristics	No.(%)
All Subjects (N = 104)	
Age years, median (range)	58 (37.25–66.75)
Gender (male/female)	51/53
NSCLC (*N* = 49)	
Age years, median (range)	64 (53–69.5)
Gender (male/female)	26/23
Pathological type	
AC	38 (73.4%)
SCC	8 (16.30%)
ASC	3 (6.1%)
Other	2 (4.1%)
TNM stage	
I	27 (55.1%)
II	2 (4.1%)
III	9 (18.4%)
IV	11 (22.5%)
Benign (*N* = 31)	
Age years, median (range)	60 (53–66)
Gender (male/female)	17/14
Healthy (*N* = 24)	
Age years, median (range)	29 (27.25–31.75)
Gender (male/female)	8/16

*NSCLC* non-small cell lung cancer; *AC* Adenocarcinoma; *SCC* Squamous carcinoma; ASC adenosquamous carcinoma

Blood samples were collected using 6 ml ACD anticoagulant vacuum vessel collection tubes for CTCs & CTECs detection and 10 ml EDTA vacuum tubes for ctDNA detection, respectively. All the blood samples were collected before either radiotherapy or chemotherapy. The collected blood samples were mixed gently to avoid any hemolysis.

Tumor DNA was extracted from the FFPE sections using the QIAamp DNA FFPE Tissue Kit (QIAGEN, CA, USA). The thickness of freshly prepared FFPE slices ranged from 5 to 10 μm. Postoperative FFPE sections with tumor cell content > 50% required 2–5 slices. The biopsy FFPE sections with tumor cell content > 20% required 5–10 pieces. After extraction, the quality of DNA was evaluated by Nanodrop One (Thermo Fisher, CA, USA), and the extracted DNA was quantified by Qubit 3.0 using the dsDNA HS Assay Kit (Life Technologies, CA, USA), according to the manufacturers’ guidelines.

### Detection and identification of CTCs and CTECs

Subtraction enrichment and immunostaining-fluorescence in situ hybridization (SE-iFISH) were applied to CTCs and CTECs detection. Firstly, target cells were enriched from 6 ml peripheral blood after the process of removing the white blood cells by centrifugation and incubation with immuno-magnetic beads, according to the manufacturer’s protocols (Cytelligen, CA, USA). Secondly, the enriched cells were stained with fluorescent-labeled antibodies, including leukocyte marker (Alexa Fluor 594-CD45, red), endothelial marker (Alexa Fluor 488/Cy5-CD31, green/gold), tumor epithelial marker (Alexa Fluor 488/Cy5-CK18, green/gold), tumor immune marker (Alexa Fluor 488-PD-L1, green), and mesenchymal marker (Cy7-Vimentin, cyan). Next, after in situ hybridization using chromosome 8 centromere (CEP8) probe (Orange), target cells were initially screened and counted by the Metafer-Image Scanning System (Carl Zeiss, Oberkochen, Germany; MetaSystems, Altlussheim, Germany; Cytelligen). After manual review, CTCs and CTECs were finally identified. All the procedures and the standard morphological criteria were detailed in our previously published article [33]. Notably, vimentin-positive cells, which were not stained in all patients, were not included in the final counts.

### ctDNA library preparation and NGS

At first, EDTA-anticoagulated blood samples were centrifuged at room temperature 200 g for 12 min, the supernatant was taken and centrifuged at room temperature 1200 g for 5 min to remove platelets and then centrifuged again at 4 °C for 16000 g for 10 min. The prepared plasma was frozen at -80 °C until further NGS library construction. Isolating cfDNA from 4 ml plasma using the AVENIO cfDNA Isolation Kit according to the manufacturer’s instructions (Roche, Mannheim, Germany). After that, the plasma-extracted cfDNA was quantified using the Qubit dsDNA HS Assay Kit with Qubit 3.0 fluorimeter (ThermoFisher Scientific, CA, USA) and qualified using High Sensitivity DNA Chip with Agilent 2100 Bioanalyzer (Agilent Technologies, CA, USA). Next, the NGS libraries were prepared with DNA input of 50 ng using the AVENIO ctDNA Library Prep kit, AVENIO ctDNA Enrichment kit, AVENIO ctDNA Expanded Panel (targeted total of 77 genes, see Additional file 3: Table S5), and AVENIO Post-Hybridization kit. After quality control (QC) of the enriched libraries, the samples were performed for pooling and sequencing with NextSeq 500/550 High Output Kit v2 (300 cycles) on the Illumina NextSeq 500 (Illumina, CA, USA).

### Tumor DNA library preparation and NGS

All samples were sequenced in a Clinical Laboratory Improvement Amendments (CLIA)- and College of American Pathologists (CAP)-certified genomic testing facility (Geneseeq Technology Inc., Nanjing, China). After being isolated from the tumor tissues, DNA fragments underwent end-repairing, A-tailing, and ligation with indexed adapters, and were selected as the size of 200 bp using Agencourt AMPure XP beads. Sequencing libraries were prepared by using the KAPA Hyper DNA Library Prep Kit (KAPA Biosystems, Wilmington, MA) according to the manufacturer’s protocol. Hybridization-based target enrichment was performed with customized xGen lockdown probes (Integrated DNA Technologies) targeting 474 pan-solid tumor-relevant genes (Geneseeq radiotron^®^, Geneseeq Technology Inc.), the list of 474 genes, see Additional file3: Table S6. Captured libraries were PCR-amplified with KAPA HiFi Hot Start Ready Mix (KAPA Biosystems Wilmington, MA) followed by quantification using the KAPA Library Quantification kit (KAPA Biosystems Wilmington, MA, USA). DNA sequencing was performed on the HiSeq4000 NGS platform (Illumina) with a paired-end 150-bp read length.

### WBCs DNA library preparation and NGS

Initially, WBCs DNA was extracted using AmoyDx Blood DNA Isolation Kit according to the manufacturer’s instructions (Amoy Diagnostics Ltd, Xiamen, China). Subsequently, the DNA derived from the WBCs was assessed for quantification using the Qubit dsDNA HS Assay Kit (ThermoFisher). Sequencing libraries were prepared by using the Homologous Recombination Repair (HRR) Related 32 Gene Library Prep Kit (Amoy) and Genetic 61 Gene Library Prep Kit (Amoy) respectively, according to the manufacturer’s protocol. The gene list of 32 Gene and 61 Gene panel were detailed in the Additional file3: Table S9, S10. After QC of the enriched libraries, the samples were performed for pooling and sequencing on the MiseqDx platform (Illumina) with a paired-end 150-bp read length.

### Bioinformatic analysis

NGS analysis pipelines were prepared for data from blood samples and corresponding tumor tissues, respectively. The FASTQ files of ctDNA were analyzed using the AVENIO ctDNA Analysis Software, specifically version 1.1.0. For the analysis of WBCs DNA, the AmoyDx gHRR Analysis Software (version 1.5.0) and AmoyDx 61Gene Analysis Software (version 0.6.2) were employed for the whole process. For tumor tissues, Trimmomatic (version 0.36) was initially performed for the FASTQ files quality control. After that, high-quality reads were aligned to the reference human genome (hg19, GRCh37) through Burrows-Wheeler aligner (BWA) v0.7.12. Deduplication was removed with Picard, and local realignment around indels and base quality score recalibration was performed with the Genome Analysis Toolkit (GATKv3.2). Furthermore, somatic single nucleotide variants (SNVs) and short insertions/deletions (indels) were identified by VarScan2, and copy number variations (CNVs) were detected using CNV Kit. CNV gain and loss were identified if the depth rate was ≥ 2.0 or ≤ 0.6 for the tissue sample. SNVs and indels were further filtered using the following criteria: (1) minimum ≥ 4 variant supporting reads and ≥ 2% variant allele frequency (VAF) supporting the variant, (2) filtered if present in > 1% population frequency in the 1000 g or ExAC database, (3) filtered through an internally collected list of recurrent sequencing errors on the same sequencing platform. The final list of mutations was annotated using vcf2maf. The mean effective coverage depth was > 3000 × for the plasma samples and > 1000 × for tumor tissues. All the somatic variants were further classified based on NCCN/AMP/ASCO/CAP guidelines.

### Meta-analysis

*Study design*: Case–control or cohort research articles published between 2019 and 2023 were included in the PubMed databases. The search terms used are shown as follows: non-small cell lung cancer, circulating tumor DNA, next generation sequencing, and tissue. The language was limited to English. The search strategy is designed as follows: (((non-small cell lung cancer[Title/Abstract]) AND (circulating tumor DNA[Title/Abstract])) AND ((next generation sequencing[Title/Abstract])) AND (tissue[Title/Abstract]))). Inclusion criteria: Research subjects: NSCLC patients; Intervention factor: NGS genotyping on ctDNA samples; Comparator factors: paired-tissue samples for genotyping; Outcome indicators: whether the ctDNA mutation profile was consistent with that in tumor tissue DNA. Exclusion criteria: review or meta-analysis or case report; repeated publications; absence of data; the research content does not align with the expectations; journal of publication not belonged to JCR partition Q1 or Q2; unpaired tissue samples. The detailed process of publications screening is presented in Additional file 1: Figure S1. *Data extraction*: Two reviewers extracted data from all eligible research, including the first author’s name, clinical stage, ctDNA method, comparator method, and targeted gene. The testing results for multiple genes including true positive (TP), false positive (FP), false negative (FN), and true negative (TN) were collected. Genomic alterations in tissue genotyping were considered the “gold standard”. All included studies underwent quality assessment using the standardized instrument Quality Assessment of Diagnostic Accuracy Tests (QUADAS-2) and summarized in Additional file 2: Figure S2.

### Statistics analysis

SPSS Statistics software (version 25.0) was performed for the data analysis. The nonparametric Mann-Whitey U test (two groups) and Kruskal–Wallis test (multiple groups) were used to compare the measurement data respectively. The inspection level was α = 0.05. ROC analysis was constructed to determine the cut-off scores for selected indicators and compare the diagnostic efficacy of selected indicators for NSCLC. Correlation analysis was performed using the nonparametric Pearson correlation. The inspection level* P *< 0.05 was defined as statistically significant. Statistical data was visualized using Prism 7.0 (GraphPad Software Inc., CA, USA) as well as OriginLab 2021 (Origin Software, MA, USA). For meta-analysis, the diagnostic data of TP, FP, FN, and TN were tabulated. The summary Receiver Operating Characteristic analysis was performed for meta-analysis of diagnostic accuracy. Software Review Manager 5.3 was used for relative analysis.

## Results

### Characterization of CTCs and CTECs in participants

**Fig. 2:**
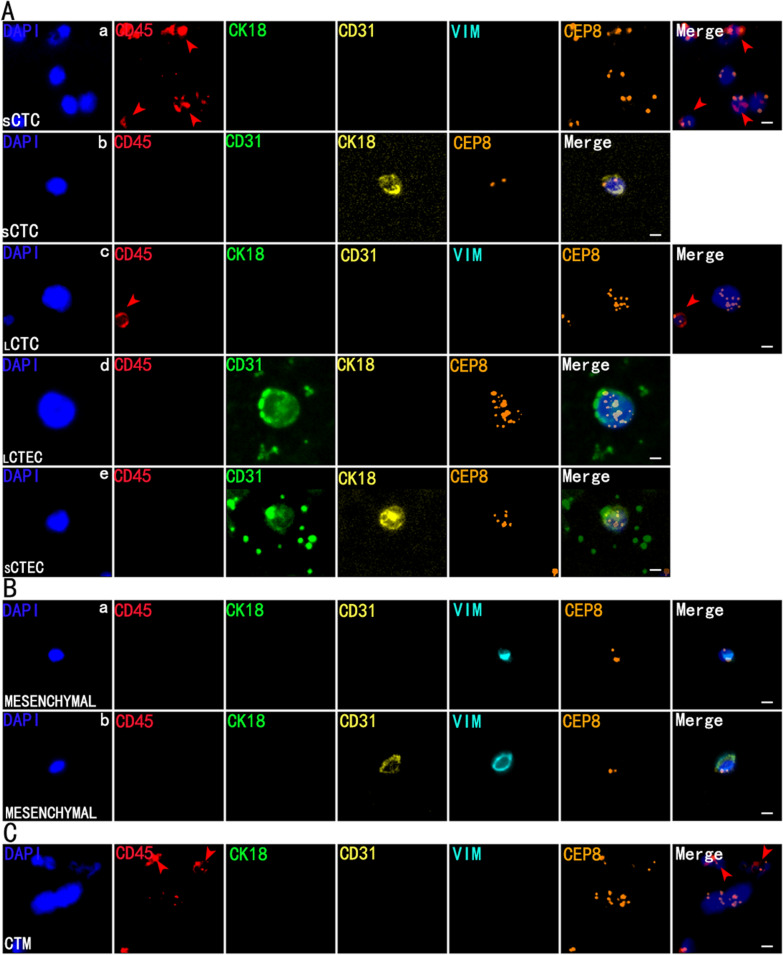
Multi-Fluorescence circulating tumor cells (CTCs) and circulating tumor-derived endothelial cells (CTECs) in patients with lung cancer: single-tumor-biomarker SE-iFISH (five/six-channel): nucleus (blue), CD45 (red), CD31 (green/yellow), CK18 (green/yellow), vimentin (cyan) and CEP8 (orange). **A** CTCs and CTECs with multiple markers in different sizes. **A**-**a** DAPI+/CD45-/CK18-/CD31-/VIM-/CEP8 small triploid CTC with adjacent WBCs (red arrows). **A**-**b** DAPI+/CD45-/CD31-/CK18+/CEP8 small diploid CTC. **A**-**c**.DAPI+/CD45-/CK18-/CD31-/VIM-/CEP8 large multiploid CTC with adjacent WBC (red arrow). **A**-**d** DAPI+/CD45-/CD31+/CK18-/CEP8 large multiploid CTEC. **A-e** DAPI+/CD45-/CD31+/CK18+/CEP8 small multiploid CTEC **B** Vimentin+ Mesenchymal cells from patients with lung squamous cell carcinoma. **B-a** DAPI+/CD45-/CK18-/CD31-/VIM+/CEP8 diploid mesenchymal cell. **B-b** DAPI+/CD45-/CK18-/CD31+/VIM+/CEP8 diploid mesenchymal cell. **C.** Circulating tumor microemboli (CTM) from patients with squamous cell carcinoma with adjacent WBCs (red arrows). Bars: 5μm

**Fig. 3:**
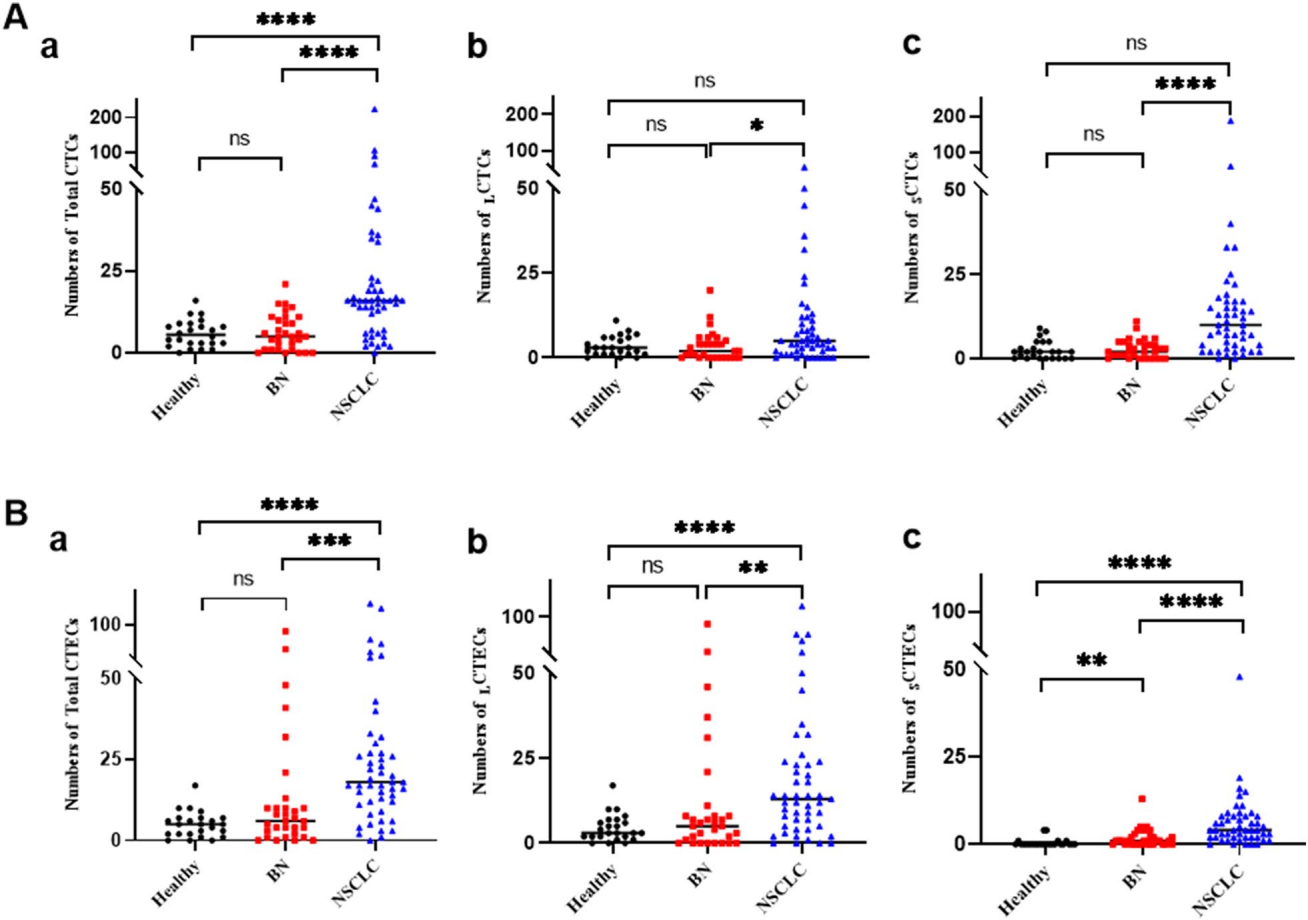
Comparison of CTCs and CTECs counts among healthy, benign lung disease (BN) and NSCLC group. **A-a** Comparison of total CTCs counts among the groups. **A-b** Comparison of large CTCs (_L_CTCs) counts among the groups. **A-c** Comparison of small CTCs (_S_CTCs) counts among the groups. **B-a** Comparison of total CTECs counts among the groups. **B-b** Comparison of large CTECs (_L_CTECs) counts among the groups. **B-c **Comparison of small CTECs (_S_CTECs) counts among the groups. Data are presented as the median, *P < 0.05, **P < 0.01, ***P < 0.001, ****P < 0.0001, Mann–Whitney *U* test

**Fig. 4:**
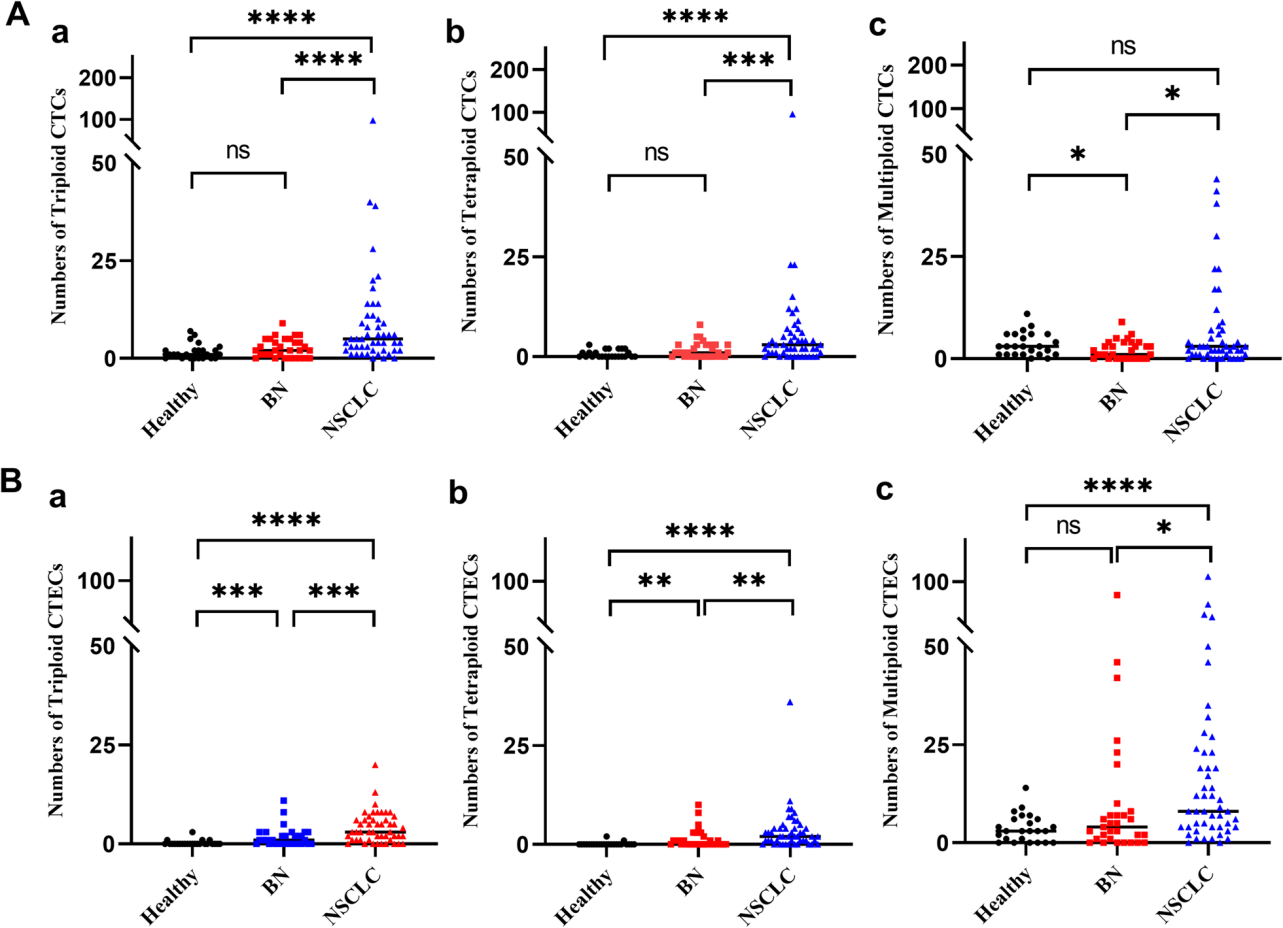
Comparison of CTCs and CTECs counts in different aneuploids among healthy, benign lung disease (BN) and NSCLC groups. **A-a** Comparison of triploid CTCs counts among the groups. **A-b** Comparison of tetraploid CTCs counts among the groups. **A-c **Comparison of multiploid CTCs counts among the groups. **B-a** Comparison of triploid CTECs counts among the groups. **B-b** Comparison of tetraploid CTECs counts among the groups. **B-c** Comparison of multiploid CTECs counts among the groups. Data are presented as the median, *P < 0.05, **P < 0.01, ***P < 0.001, ****P < 0.0001, Mann–Whitney *U* test

A variety of CTCs and CTECs with different markers were observed. CK18 negative CTCs were detected in 97.9% (48/49), 83.9% (26/31), and 95.8% (23/24) in the NSCLC, benign lung disease, and healthy control, respectively. Remarkably, CK18-positive CTCs were detected in two NSCLC patients, with a tumor marker-positive detection rate of 4.2% (2/48). The circulating tumor microemboli (CTM) were detected in 4 lung cancer patients, while not in the other groups. Similarly, CTECs were detected in almost all participants. Vimentin-positive cells and CTM were detected in three squamous cell carcinama (SCC) patients. Several typical cells identified are photographed and listed, see Fig. 2. Compared with the benign group, the CTCs and CTECs counts are 5 units/6 ml (median, 1-11) and 5 units/6 ml (median, 2-7), respectively. The CTCs and CTECs counts of NSCLC patients were significantly increased, being 16 units/6 ml (median, 7–22.5) and 18 units/6 ml (median, 11.5–28.5), respectively (Additional file 3: Table S1; *P* < 0.001, *P* < 0.001, Fig. 3A-a, Fig. 3B-a). Additionally, the number of CTCs or CTECs was also higher in different sizes or different karyotypes of the lung cancer group compared to the benign group. (ALL: *P* < 0.05, Fig. 3A-b,c and B-b,c; Fig. 4A and B; Additional file 3: Table S2).

### Diagnostic efficacy of CTCs and CTECs in NSCLC

To investigate the diagnostic efficacy of CTCs and CTECs in NSCLC, ROC analysis was performed based on multiple indicators of CTCs and CTECs using benign lung diseases as control. To illustrate the value of CTCs and CTECs for NSCLC, we selected three serum tumor markers (SCC, CEA, and CYFRA 21-1) as control, and any one of them higher than the cut-off value was considered positive. It was shown that the sensitivity and specificity of total CTCs and CTECs in the diagnosis of NSCLC were 67.3% and 77.6% [AUC (95%CI): 0.815 (0.722–0.907)], 83.9% and 77.4% [AUC (95%CI): 0.739 (0.618–0.860)], respectively, which were superior to serum tumor biomarkers or imaging evaluation (Tables 2, 3). Remarkably, small CTCs and triploid CTCs had high specificity in the diagnosis of NSCLC, which were 93.5% and 96.8% respectively. CTCs and CTECs with different sizes and karyotype have varying diagnostic sensitivity for different pathological types and stages of non-small cell lung cancer (detailed in Additional file 3: Table S3, S4). Furthermore, paired-wise combinations between CTCs and CTECs subgroups were conducted based on morphology and karyotype. After combination, the area under the ROC curves increased to Total(CTCs + CTECs) 0.826, (_S_CTCs + _S_CTECs) 0.898, and triploid(CTCs + CTECs) 0.872, respectively, indicating that the combined indicators were more effective than the single one (Table 2, Table 3).

**Table 2:** Statistical parameters for ROC analysis of CTCs and CTECs for NSCLC

Test item	AUC	Cutoff value	Std. Error	P	95% CI
Total CTCs	0.815	11.5	0.047	< 0.0001	0.722–0.907
_L_CTCs	0.663	6.5	0.061	0.015	0.544–0.782
_S_CTCs	0.815	6.5	0.046	< 0.0001	0.724–0.906
Triploid CTCs	0.809	6.5	0.047	< 0.0001	0.717–0.901
Tetraploid CTCs	0.698	4.5	0.056	< 0.001	0.634–0.852
Multiploid CTCs	0.646	6.5	0.062	0.029	0.525–0.766
Total CTECs	0.739	10.5	0.062	< 0.001	0.618–0.860
_L_CTECs	0.691	8.5	0.064	0.004	0.566–0.815
_S_CTECs	0.781	2.5	0.053	< 0.0001	0.679–0.884
Triploid CTECs	0.716	3.5	0.058	0.001	0.602–0.830
Tetraploid CTECs	0.677	2.5	0.063	0.008	0.555–0.799
Multiploid CTECs	0.675	7.5	0.063	0.009	0.550–0.799
Total CTCs + CTECs	0.826	–	0.047	< 0.0001	0.734–0.917
_S_CTCs + _S_CTECs	0.898	–	0.036	< 0.0001	0.828–0.968
Triploid(CTCs + CTECs)	0.872	–	0.040	< 0.0001	0.794–0.950

Large CTCs, _L_CTCs, small CTCs, _S_CTCs, Large CTECs, _L_CTECs, small CTECs, _S_CTECs

**Table 3:** Evaluation of the diagnostic efficiency of single and combined tests for NSCLC [%]

Test item	SEN%	SPE%	PPV%	NPV%	AC%	LR +
Total CTCs	67.3	83.9	86.8	61.9	73.8	4.2
_L_CTCs	38.8	67.7	65.5	41.2	50.0	1.2
_S_CTCs	65.3	93.5	94.1	63.0	76.3	10.1
Triploid CTCs	57.1	96.8	96.6	58.8	72.5	17.7
Tetraploid CTCs	51.0	87.1	86.2	52.9	65.0	4.0
Multiploid CTCs	28.6	96.8	93.3	46.2	55.0	8.9
Total CTECs	77.6	77.4	84.4	68.6	77.5	3.4
_L_CTECs	67.3	67.7	76.7	56.8	67.5	2.1
_S_CTECs	67.3	77.4	82.5	60.0	71.3	3.0
Triploid CTECs	49.0	90.3	88.9	52.8	65.0	5.1
Tetraploid CTECs	36.7	71.0	66.7	41.5	50.0	1.3
Multiploid CTECs	59.2	74.2	78.4	53.5	65.0	2.3
SCC + CEA + CYFRA 21–1	52.2	75.8	77.4	50.0	61.3	2.17
CT/PET-CT diagnosis	57.1	77.4	80.0	53.3	65.0	2.48

Large CTCs, _L_CTCs; small CTCs, _S_CTCs; Large CTECs, _L_CTECs; small CTECs, _S_CTECs

*SEN* Sensitivity; *SPE* Specificity; *PPV* Positive predictive value; *NPV* Negative predictive value; LR*+* Positive likelihood ratio; *SCC* Squamous cell carcinoma antigen; *CEA* Carcinoma embryonic antigen; *CYFRA21-1* Cytokeratin fragment antigen 21–1

### Cell-free DNA level in NSCLC patients with high CTCs and CTECs counts

**Fig. 5:**
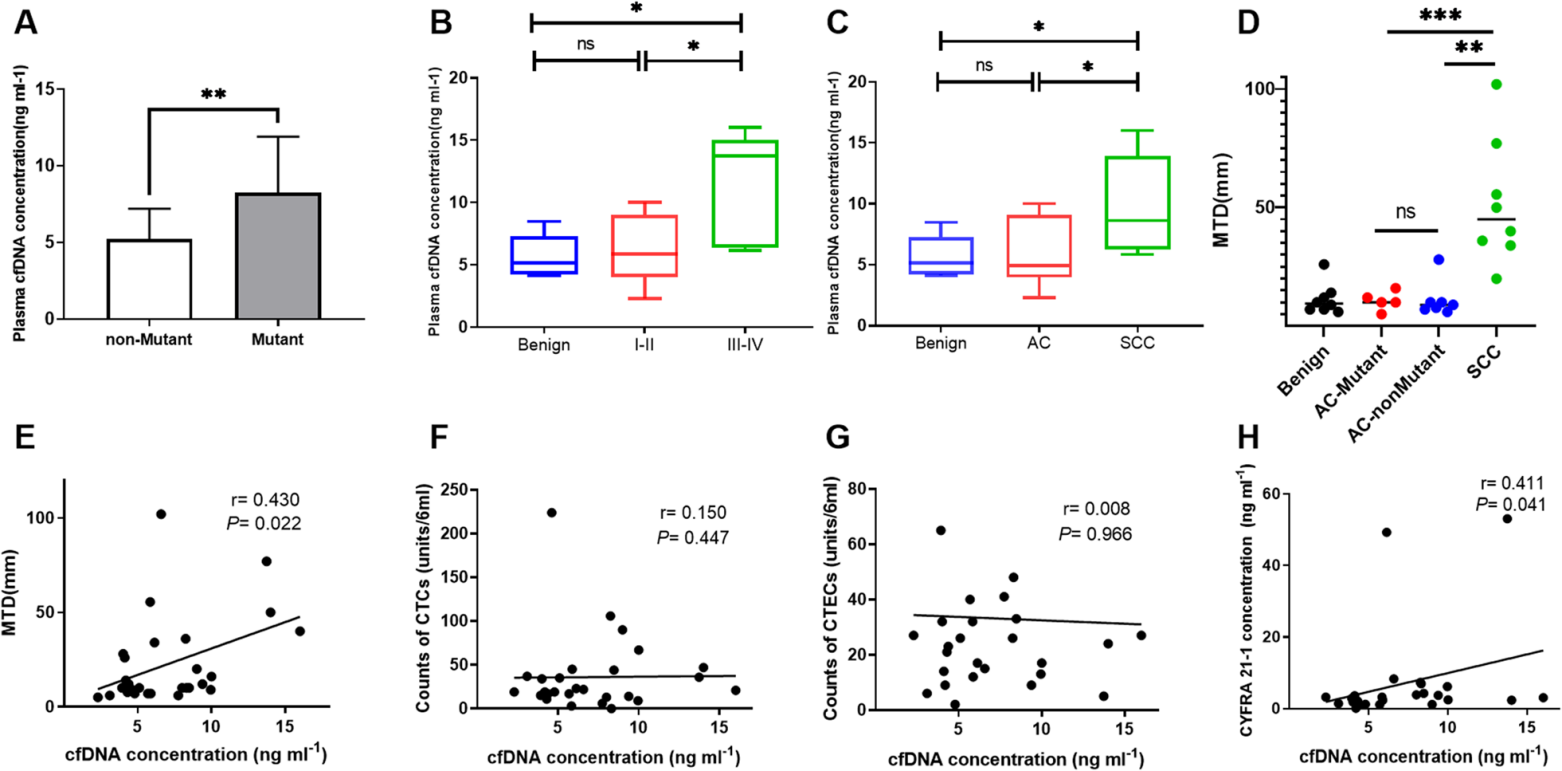
Analysis of the plasma cfDNA concentration in enrolled 28 patients. **A** Plasma cfDNA concentration between patients with or without ctDNA mutations**. B**  Plasma cfDNA concentration between different TNM stages. **C** Plasma cfDNA concentration between different pathological types. **D** Tumor size (Maximum tumor diameter, MTD) between different pathological types. **E** The correlation between cfDNA concentration and tumor size (MTD). **F** The correlation between cfDNA concentration and counts of CTCs. **G** The correlation between cfDNA concentration and counts of CTECs. **H** The correlation between the concentration of cfDNA and CYFRA 21–1.*P < 0.05, **P < 0.01, ***P < 0.001, ****P < 0.0001, Mann–Whitney *U* test

The concentration of cell-free DNA was 7.64 ± 3.79 ng.ml^−1^ in NSCLC patients (n = 20), compared with 5.64 ± 1.67 ng.ml^−1^in the benign group (n = 8) (Table 4). Interestedly, ctDNA mutations were detected in patients with a significantly higher concentration of cfDNA (Fig. 5A). Remarkably, cfDNA was significantly higher in the III-IV stage group as well as the squamous cell carcinoma group (Fig. 5B, C). Furthermore, we conducted a thorough analysis of the correlation between cfDNA concentration and counts of CTCs and CTECs, as well as the concentration of serum biomarkers. Our findings indicated no linear correlation between different groups, such as between cfDNA concentration and CTCs counts (r = 0.150, *P* = 0.447), cfDNA concentration and CTECs counts (r = 0.008, *P* = 0.966), cfDNA and SCC concentration (r = 0.055, *P* = 0.800), and cfDNA and CEA concentration (r = − 0.211, *P* = 0.323). However, we did observe a positive correlation between cfDNA level and tumor size or maximum tumor diameter (MTD) (r = 0.430, *P* = 0.022), as well as CYFRA 21–1 concentration (r = 0.411, *P* = 0.041), (Fig. 5E, F, G, H) .

**Table 4:** Statistics of the cfDNA concentration in enrolled 28 patients (ng/ml)

Group	No(%)	Mean	S.D	95%CI	Median	Range	P25-P75
Benign	8	5.64	1.67	4.11–8.47	5.16	4.36	4.24–7.28
NSCLC	20	7.64	3.79	4.79–9.38	7.29	13.71	4.42–9.81
Pathological type							
AC	12(60%)	6.10	2.83	4.30–7.89	4.95	7.71	3.95–9.11
SCC	8(40%)	9.94	4.03	6.57–13.31	8.62	10.16	6.24–13.93
TNM stage							
I-II	15(75%)	6.42	2.67	4.94–7.89	5.84	7.71	4.01–9.00
III-IV	5(25%)	11.29	4.59	5.59–16.99	13.73	9.87	6.36–15.00

*NSCLC* non-small cell lung cancer; *AC* Adenocarcinoma; *SCC* Squamous carcinoma

### Comparison of mutational profiles between ctDNA and tumor DNA

**Fig. 6:**
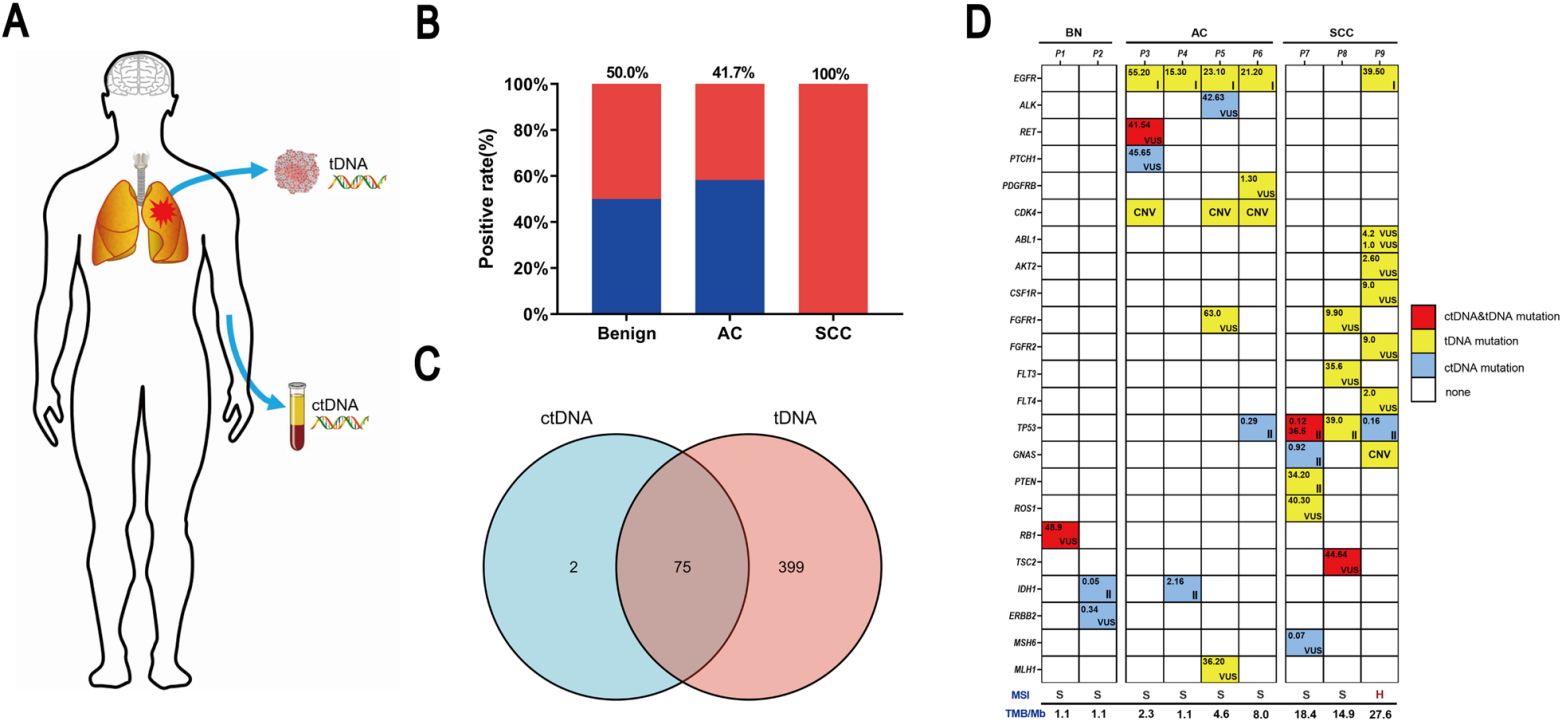
Characteristics of the mutant genes detected in circulating tumor DNA (ctDNA) and tumor DNA (tDNA). **A** Schematic figure illustrating the sources of ctDNA and tDNA. **B** The positive rates of ctDNA mutations among benign, adenocarcinoma (AC), and squamous cell carcinoma (SCC) subgroups of the enrolled 28 patients. **C** Venn figure showing the target genes covered in panels of both ctDNA and tDNA. **D** Heatmap detailing the mutant genes detected in the ctDNA and matched tDNA of the 9 patients with fractional abundance in the upper left corner as well as the microsatellite instability (MSI) status and tumor mutation burden (TMB) score; VUS: variant of uncertain significance

The ctDNA mutations were detected in 17 out of the 28 patients (60.71%), including 4 in the benign group (50.00%), 5 in the lung adenocarcinoma (AC) group (41.70%), and 8 in the SCC group (100%) (Fig. 6B). Among ctDNA mutations, the TP53 mutation was the most common mostly appearing in the SCC group (see Additional file 3: Table S7).

A total of 5 mutations were found in 4 benign patients, 3 of them belonged to Class III variants: *RB1*(p.Arg621His), 48.9%; *ERBB2* (p.Lys937Arg), 0.34%; *MET* (p.Arg1005Gly), 0.09% and 2 were Class II mutations: *IDH1* (p.Arg132Ser), 0.05%; *PTEN* (p.Asn323fs), 0.12%. Since these 5 mutations were found in patients with benign lesions, we validated these mutations using WBCs DNA from corresponding patients and found *RB1* gene mutation (p.Arg621His, 48.9%) presented in WBCs DNA (see Additional file 3: Table S11). The remaining 4 mutations, varying from 0.05% to 0.34%, were not detected in WBCs DNA and suggested they were somatic mutations. It is worth mentioning that the patient with *PTEN* (p.Asn323fs) was suspected to be malignant based on imaging examinations, even though his bronchial biopsy was diagnosed as benign.

In order to validate the mutations, found in ctDNA, also presented in tDNA, NGS was performed in matched tDNA from 9 patients (2 with benign lesion, 4 with adenocarcinoma, 3 with SCC). The panel for tDNA contained 474 pan-solid tumor-relevant genes, which covered 75 genes in the ctDNA panel except 2 genes *CCND2 *and *CCND3* (Fig. 6C). The full gene list for both panels is shown in Additional file 3: Table S5 and Table S6. In these 9 patients, a total of 92 tDNA mutations were detected and 26 of them occurred in overlapping genes (see Additional file 3: Table S8). In detail, 1 mutation in 1 case of the benign lesion (another case of the benign lesion had no mutation detected), 11 mutations in 3 cases of AC, and 14 mutations in 3 cases of SSC (detailed mutation and frequency shown in Fig. 6D). The most common mutation was *EGFR* p.﻿L﻿﻿85﻿8R and it was observed in 4 patients with AC and in 1 patient with SCC, but it was not detected in cfDNA. The second common mutation was CNV involved in *CDK4*. It was surprising that only 4 mutations *RB1* (p.R621H), *RET* (p.D631N), *P53* (p.V216L), and *TSC2* (p.R718H) were detected in both the tDNA and ctDNA. It suggested that the a lower coincidence among ctDNA and tDNA mutations. However, *PTCH1* (c.1347 + 6G > A) and *ALK* (Ser691Ser), which had a high frequency of 45.2% and 42.63% in plasma respectively, were not detected in the matched tumor tissue (Fig. 6D). Another interesting point is that tumor mutation burden (TMB) was higher in the SCC group and microsatellite instability-high (MSI-H) status was also observed in 1 SCC patient.

**Fig. 7:**
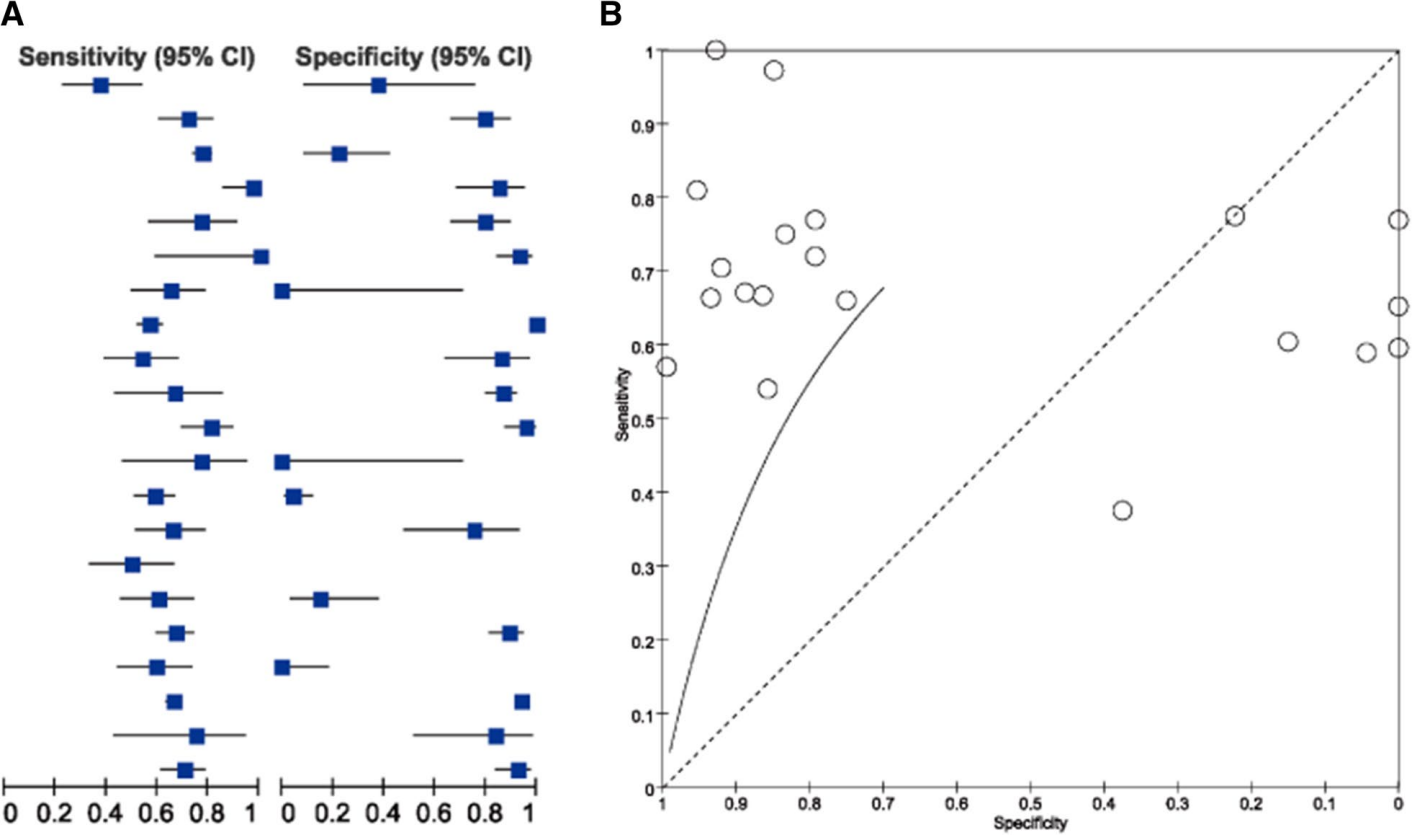
Meta-Analysis of results for the ctDNA genotyping vs tissue genotyping of 21 studies. **A** Forest plot of the analysis for the ctDNA genotyping. **B** sROC analysis for the ctDNA genotyping

Since the sample size in our study is relatively small, we performed an additional meta-analysis from 21 articles containing ctDNA and tDNA sequencing data. The sensitivity, specificity, staging as well as methodology of each article are shown in Table 5. The rate of mutation to be detected or the sensitivity of mutation detection was different depending on the clinic staging. Among several studies involving advanced patients, the sensitivity ranged from 50 to 100% [34–53], However, the sensitivity was as low as 37% in studies focusing on early-stage patients [54] (Fig. 7A, Table 5). Moreover, the size of the panel used for detection also had an impact on the sensitivity. In two studies [40, 46], where only the *EGFR* or *KRAS* gene was covered, the sensitivity was significantly higher compared to the first study in the list [54], which used a broader panel of genes (Table 5).

**Table 5:** Characteristics of included studies for meta-analysis

Study	Stage	TP	FP	FN	TN	Sensitivity (95% CI)	Specificity (95% CI)	ctDNA Method	tDNA Method	Targeted Gene
Bin Zhang et al	early-stage	15	5	25	3	0.38 (0.23–0.54)	0.38 (0.09–0.76)	NGS	NGS	Panel Targeted 546 GENES*
Charu Aggarwal et al	IV	54	11	21	42	0.72 (0.60–0.82)	0.79 (0.66–0.89)	NGS	NGS	Panel Targeted 73 GENES;153GENES*
Chenglong Zhao et al	Advanced	381	21	111	6	0.77 (0.73–0.81)	0.22 (0.09–0.42)	NGS	NGS	Panel Targeted 168 GENES
García-Pardo et al	Advanced	35	5	1	28	0.97 (0.85–1.00)	0.85 (0.68–0.95)	NGS	NGS	EGFR; ALK;ROS1;MET;ERBB2;BRAF;KRAS
Hao Bai et al	I-IV	20	11	6	42	0.77 (0.56–0.91)	0.79 (0.66–0.89)	NGS	AMRS-PCR	EGFR
Jian-xin Yin et al	IA-IV	7	5	0	64	1.00 (0.59–1.00)	0.93 (0.84–0.98)	NGS	NGS	EGFR; KRAS
Jun Cai et al	Advanced	30	3	16	0	0.65 (0.50–0.79)	0.00 (0.00–0.71)	NGS	NGS	Panel Targeted 95 GENES*
Justin Jee et al	Advanced	216	3	163	538	0.57 (0.52–0.62)	0.99 (0.98–1.00)	NGS	NGS	Panel Targeted 24GENES*
Lawrence Hsu Lin et al	II- IV	27	3	23	18	0.54 (0.39–0.68)	0.86 (0.64–0.97)	NGS	NGS	Panel Targeted 74 GENES*
Luis E. Raez et al	IV	14	18	7	114	0.67 (0.43–0.85)	0.86 (0.79–0.92)	NGS	NGS	Panel Targeted 86 GENES*
Maria Gabriela O. et al	II IV	51	3	12	61	0.81 (0.69–0.90)	0.95 (0.87–0.99)	NGS	NGS	Panel Targeted 24 GENES*
Martin Metzenmacher et al	IV	10	3	3	0	0.77 (0.46–0.95)	0.00 (0.00–0.71)	NGS	NGS	Panel Targeted 73 GENES*
Moom R. et al	I-IV	89	66	62	3	0.59 (0.51–0.67)	0.04 (0.00–0.12)	NGS	NGS	ALK,EGFR,ROS1,BRAF,RET,MET, ERBB2, KRAS
Paul van et al	Advanced	33	4	17	12	0.66 (0.51–0.79)	0.75 (0.48–0.93)	NGS	NGS	EGFR, KRAS, BRAF, ERBB2, and PIK3CA
Peng-Peng Kuang et al	IB-III	19	0	19	0	0.50 (0.33–0.67)	Not estimable	NGS	NGS	Panel Targeted 425 GENES*
Ramon Palmero et al	Advanced	29	17	19	3	0.60 (0.45–0.74)	0.15 (0.03–0.38)	NGS	NGS	ALK,EGFR,ROS1,BRAF,RET, MET, ERBB2, KRAS
Sehhoon Park et al	Advanced	108	11	53	87	0.67 (0.59–0.74)	0.89 (0.81–0.94)	NGS	NGS	Panel Targeted 74 GENES*
Sheehyun Kim et al	Advanced	28	18	19	0	0.60 (0.44–0.74)	0.00 (0.00–0.19)	NGS	NGS	Panel Targeted 171 and 25 fusion genes*
Vassiliki A. et al	Advanced	566	53	287	754	0.66 (0.63–0.70)	0.93 (0.91–0.95)	NGS	PCR	EGFR
Yukti Choudhury et al	I-IV	9	2	3	10	0.75 (0.43–0.95)	0.83 (0.52–0.98)	NGS	NGS	Panel Targeted 49 GENES*
Zeyun Lin et al	I-IV	81	6	34	69	0.70 (0.61–0.79)	0.92 (0.83–0.97)	NGS	NGS	Panel Targeted 168 GENES*

*EGFR* epidermal growth factor receptor; *ALK* anaplastic lymphoma kinase; *ROS1* *ROS* proto-oncogene 1, receptor tyrosine kinase; *HER2/ERBB2* human epidermal growth factor receptor 2; *BRAF* serine/threonine-protein kinase B-Raf; *MET* MET proto-oncogene, receptor tyrosine kinase; *RET* *RET* proto-oncogene; *KRAS* proto-oncogenes, *K-rasFN*, false negative; *FP* false positive; *TP* true positive; *TN* true negative;  *NGS* next generation sequencing

^*^The genes panels some studies applied include 8 core driver genes in lung cancer: EGFR、ALK、ROS1、BRAF、RET、MET、HER2、KRAS

## Discussion

Our study's findings indicated that CTCs and CTECs are valuable in aiding the diagnosis of lung cancer. Furthermore, our recently published study provides more comprehensive results, including patients with solitary pulmonary nodules, which further confirms the diagnostic value of this liquid biopsy method for early-stage lung cancer [33]. Therefore, in this article, we mainly discuss the role of ctDNA detection for early diagnosis and the coincidence between ctDNA mutation and tDNA mutation in order to suitably use it in clinical.

It is well known that cfDNA can be rapidly cleared in circulation, and it is difficult to capture due to low concentration and a very short half-life of less than one hour. Additionally, the proportion of ctDNA in cfDNA undergoes a profound alteration as cancer advances, ranging from 0.1 to 90% [55]. Thus, high sensitivity and stable assays are important for both cfDNA isolation and ctDNA mutation profiling. Currently, multiple methods have been applied for ctDNA mutation detection, such as ddPCR, BEAMing, Tagged-Amplicon deep sequencing (TAm-seq), Cancer Personalized Profiling by deep sequencing (CAPP-Seq), and Whole-genome-sequencing (WGS) or Whole-exome sequencing (WES). Compared to PCR-based assays, which can only detect a limited number of mutations, NGS-based methods utilizing unique molecular barcodes can identify and quantify multiple target genes simultaneously. This allows for the detection of mutant allele fractions (MAF) as low as < 0.1% [56]. In our research, we applied the Roche AVENIO ctDNA Expanded Kit and Illumina NextSeq 500 platform to detect ctDNA mutations in targeted 77 genes. Since low-abundance ctDNA mutations, such as *ERBB2* and *IDH1* with the percentage of variants 0.34% and 0.05% respectively, had been caught in one patient with the benign lesion, which suggested that this kit is relatively sensitive and NGS-based ctDNA detection may have advantages in early finding mutant ctDNA.

In this study, even though the cell-free DNA concentration showed a significant positive association with the tumor size and stage, we still cannot confirm how much of this cfDNA is derived from the tumor tissue, since the cfDNA level can also increase in many instances such as inflammation, surgery or drug stimulation which could cause cfDNA release by normal cells or hematopoietic cells [32]. In addition, we found no correlation between cfDNA concentration and CTCs or CTECs counts, which does bolster the previous research that most cfDNA is not derived from circulating tumor cells [57].

One unanticipated finding in this research was the discordance in mutations between ctDNA from blood and DNA derived from matched tumor tissue. On the one hand, low-frequency variants such as *I﻿DH1* (Arg13﻿﻿2G﻿ly﻿), *TP53 *(c﻿.﻿﻿919 + 1G > ﻿A﻿), *GNAS* (Ar﻿g201Cys) and *MSH *(Try1066Cys), as well as high-frequency variants  *PTCH1*(c.1347 + 6G > A) and *ALK* (Ser691Ser) detected in the plasma of patients were not found in the matched tumor. We considered that those low-frequency variants may arise from minor subclones due to temporal and spatial heterogeneity of the tumor itself [58], while the high-frequency mutations may be related to clonal hematopoiesis (CH) [30, 59–61]. On the other hand, many mutations in tumor tissue especially for *EGFR* (L858R) have not been detected in the plasma. This may be possibly due to the minimal release of early-stage tumors. Unlike CTCs that are actively discharged from the tumor tissues and can be viewed as small metastases, known as cM0(i +) staging [62], ctDNA is released passively by apoptotic and necrotic cancer cells. Therefore, external factors like surgery, radiation, or chemotherapy have the potential to promote the release of tumor DNA into the bloodstream. Additionally, tumor heterogeneity may also be attributed to this concordance. Our meta-analysis results also show the sensitivity of ctDNA testing using NGS is higher in advanced NSCLC but not satisfactory in early-stage patients, which is similar to our findings. Thus, it means that a high-intensity ctDNA assay is needed to capture these rare mutations.

## Conclusions

In summary, the detection of CTCs and CTECs based on SE-iFISH has shown promising roles for NSCLC diagnosis. However, ctDNA detection provides an option for screening potential patients with cancer and monitoring treatment response. Despite liquid biopsy shows currently various dilemmas in clinical practice, with the progress of technology, the reduction of sequencing costs as well as big data analysis based on multiple indicators, it will eventually play a more important role in the diagnosis, treatment, or prognosis of various cancers in the future.

### Supplementary Information

**Additional file 1: Fig S1.** Flowchart of publications selection in meta-analysis.

**Additional file 2: Fig S2.** Included studies quality assessment according to QUADAS-2. **A.** Risk of bias and applicability concerns summary: review authors' judgments about each domain for each included study **B.** Risk of bias and applicability concerns graph.

**Additional file 3:** Supplementary **Table 1.** Statistical parameters of CTCs and CTECs of 104 participating subjects; **Table 2.** Statistical parameters of aneuploid CTCs and CTECs of 104 participating subjects; **Table 3.** The diagnostic sensitivity of CTCs and CTECs in different pathological types and stages of NSCLC; **Table 4.** The diagnostic sensitivity of aneuploid CTCs and CTECs in different pathological types and stages of NSCLC; **Table 5.** Target gene information of ctDNA panel; **Table 6.** Target gene information of tDNA panel; **Table 7.** Mutational profile of ctDNA in enrolled 28 patients; **Table 8.** Mutational profiles of tumor DNA in filtered 9 patients; **Table 9.** Target gene information of HRR panel; **Table 10.** Target gene information of 61 GENE panel; **Table 11.** Mutational profiles of WBCs DNA in benign 4 patients.

## Data Availability

All data generated during this study are available from the corresponding author by request.

## Abbreviations

AC: Adenocarcinoma
CTCs: Circulating tumor cells
CTECs: Circulating tumor-derived endothelial cells
ctDNA: Circulating tumor DNA
NGS: Next generation sequencing
NSCLC: Non-small cell lung cancer
SCC: Squamous cell carcinoma
SE-iFISH: Subtraction enrichment and immunostaining-fluorescence in situ hybridization
tDNA: Tumor DNA
