# Supplementary figures and images for: A comparative study on ctDNA and tumor DNA mutations in lung cancer and benign cases with a high number of CTCs and CTECs
Source: J Transl Med. 2023 Dec 1;21:873. doi: 10.1186/s12967-023-04746-8 (PMC10691057; doi:10.1186/s12967-023-04746-8)

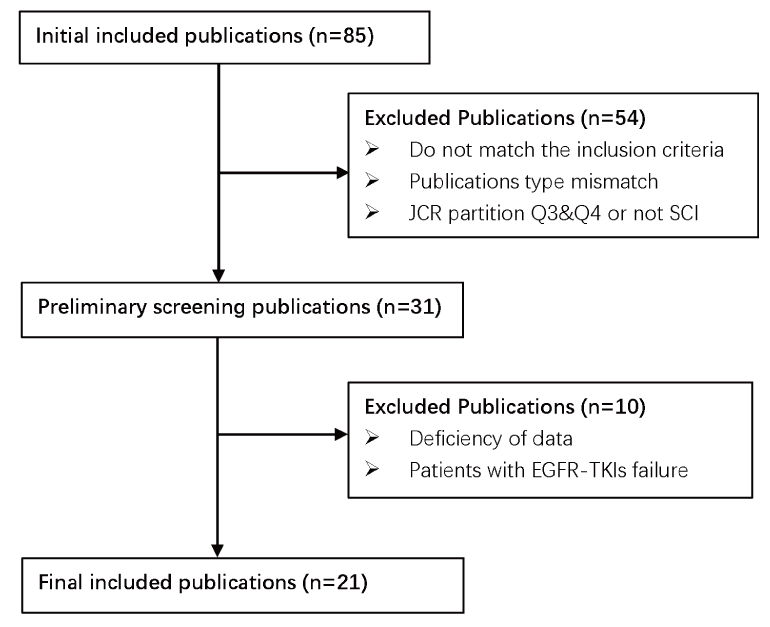

Supplement: Supplementary file 1 — Additional file 1: Fig S1. Flowchart of publications selection in meta-analysis. [file 12967_2023_4746_MOESM1_ESM.tif]

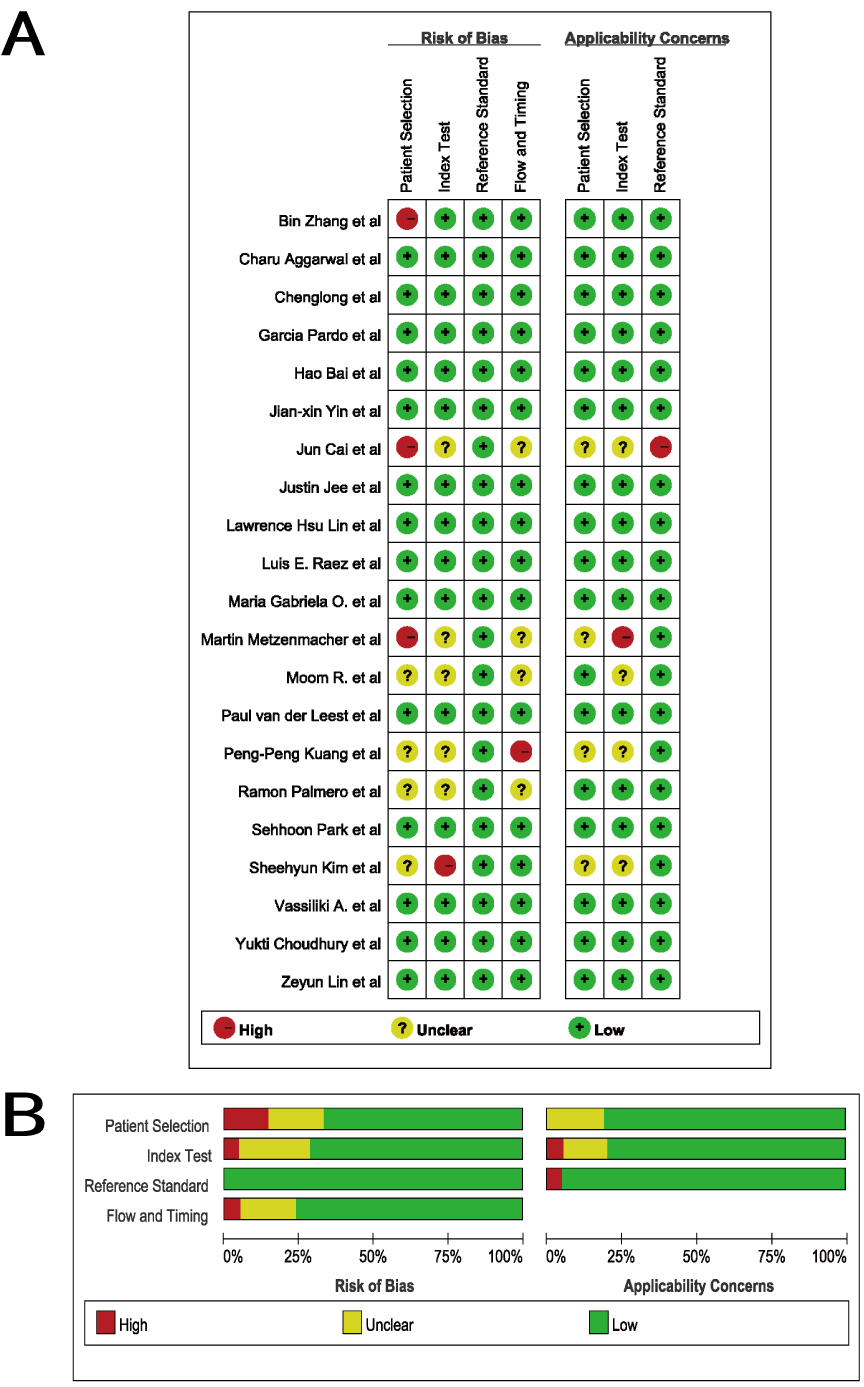

Supplement: Supplementary file 2 — Additional file 2: Fig S2. Included studies quality assessment according to QUADAS-2. A. Risk of bias and applicability concerns summary: review authors' judgments about each domain for each included study B. Risk of bias and applicability concerns graph. [file 12967_2023_4746_MOESM2_ESM.tif]
