# Supplementary material for: A comparative study on ctDNA and tumor DNA mutations in lung cancer and benign cases with a high number of CTCs and CTECs
Source: J Transl Med. 2023 Dec 1;21:873. doi: 10.1186/s12967-023-04746-8 (PMC10691057; doi:10.1186/s12967-023-04746-8)
Supplement: Supplementary file 3 — Additional file 3: Supplementary Table 1. Statistical parameters of CTCs and CTECs of 104 participating subjects; Table 2. Statistical parameters of aneuploid CTCs and CTECs of 104 participating subjects; Table 3. The diagnostic sensitivity of CTCs and CTECs in different pathological types and stages of NSCLC; Table 4. The diagnostic sensitivity of aneuploid CTCs and CTECs in different pathological types and stages of NSCLC; Table 5. Target gene information of ctDNA panel; Table 6. Target gene information of tDNA panel; Table 7. Mutational profile of ctDNA in enrolled 28 patients; Table 8. Mutational profiles of tumor DNA in filtered 9 patients; Table 9. Target gene information of HRR panel; Table 10. Target gene information of 61 GENE panel; Table 11. Mutational profiles of WBCs DNA in benign 4 patients. [file 12967_2023_4746_MOESM3_ESM.doc]

Supplemental Table 1. Statistical parameters of CTCs and CTECs of 104 participating subjects

|  | **CTCs (Units/6ml)** | | | | | | | | | | | | | | | | | | | | | |  | **CTECs (Units/6ml)** | | | | | | | | | | | | | | | | | |
| --- | --- | --- | --- | --- | --- | --- | --- | --- | --- | --- | --- | --- | --- | --- | --- | --- | --- | --- | --- | --- | --- | --- | --- | --- | --- | --- | --- | --- | --- | --- | --- | --- | --- | --- | --- | --- | --- | --- | --- | --- | --- |
| **Characteristics** | **Total CTCs** | | | | | | |  | **_L_CTCs** | | | | | |  | **sCTCs** | | | | | | |  | **Total CTECs** | | | | | |  | **_L_CTECs** | | | | | |  | **sCTECs** | | | |
|  | **M** | | **P25-P75** | | | ***P*** | |  | **M** | | **P25-P75** | | | ***P*** |  | M | | **P25-P75** | | | ***P*** | |  | **M** | | **P25-P75** | | | ***P*** |  | **M** | | **P25-P75** | | | ***P*** |  | **M** | | **P25-P75** | ***P*** |
| Healthy | 5.5 | | 3-8.75 | | | <0.001 | |  | 3 | | 1-6 | | | 0.027 |  | 2 | | 0-4.5 | | | <0.0001 | |  | 6 | | 2-10 | | | <0.001 |  | 3 | | 2-6.75 | | | 0.0001 |  | 0 | | 0-0 | <0.0001 |
| BLDP | 5 | | 1-11 | | |  | |  | 2 | | 0-5 | | |  |  | 2 | | 0-5 | | |  | |  | 5 | | 2-7 | | |  |  | 5 | | 0-8 | | |  |  | 1 | | 0-2 |  |
| NSCLC | 16 | | 7-22.5 | | |  | |  | 5 | | 1.5-11.5 | | |  |  | 10 | | 3.5-17 | | |  | |  | 18 | | 11.5-28.5 | | |  |  | 13 | | 5-24 | | |  |  | 4 | | 2-8 |  |
| Pathological type | |  | |  |  | | 0.629 | | |  | |  |  | 0.153 | | |  | |  |  | | 0.891 | | |  | |  |  | 0.183 | | |  | |  |  | 0.557 | | | 0.156 | | |
| AC | 16 | | 8.25-21.75 | | |  | |  | 4 | | 2-10.75 | | |  |  | 10.5 | | 3.25-17 | | |  | |  | 17 | | 9.5-27 | | |  |  | 14 | | 5-23.75 | | |  |  | 4 | | 1-1.75 |  |
| SC | 14 | | 5.5-37.75 | | |  | |  | 7.5 | | 2.25-26 | | |  |  | 8 | | 4.75-12 | | |  | |  | 20 | | 13.25-36.5 | | |  |  | 12 | | 4.5-30 | | |  |  | 5 | | 3-8.75 |  |
| ASC | 34 | | 3-90 | | |  | |  | 16 | | 2-50 | | |  |  | 18 | | 1-40 | | |  | |  | 32 | | 22-131 | | |  |  | 18 | | 13-115 | | |  |  | 16 | | 4-19 |  |
| Other | 9 | | 2-16 | | |  | |  | 0.5 | | 0-1 | | |  |  | 8.5 | | 2-15 | | |  | |  | 10.5 | | 6-15 | | |  |  | 8 | | 2-14 | | |  |  | 2.5 | | 1-4 |  |
| TNM stage |  | |  | | | 0.715 | |  |  | |  | | | 0.747 |  |  | |  | | | 0.564 | |  |  | |  | | | 0.388 |  |  | |  | | | 0.525 |  |  | |  | 0.826 |
| Ⅰ | 16 | | 6-23 | | |  | |  | 4 | | 1-12 | | |  |  | 13 | | 2-17 | | |  | |  | 17 | | 8-30 | | |  |  | 11 | | 4-26 | | |  |  | 4 | | 2-8 |  |
| Ⅱ | 36 | | 5-67 | | |  | |  | 27.5 | | 1-54 | | |  |  | 8.5 | | 4-13 | | |  | |  | 18 | | 17-19 | | |  |  | 14 | | 12-16 | | |  |  | 4 | | 1-7 |  |
| Ⅲ | 14 | | 7.5-17 | | |  | |  | 5 | | 1.5-8.5 | | |  |  | 7 | | 3-9.5 | | |  | |  | 17 | | 7-23.5 | | |  |  | 12 | | 1.5-19 | | |  |  | 4 | | 0.5-8.5 |  |
| Ⅳ | 16 | | 14-34 | | |  | |  | 5 | | 4-16 | | |  |  | 12 | | 8-18 | | |  | |  | 25 | | 16-73 | | |  |  | 18 | | 9-67 | | |  |  | 6 | | 2-8 |  |

Measurement data were compared between two groups by Mann-Whitney U test, multiple groups by Kruskal-Wallis test. Abbreviations: NSCLC non-small cell lung cancer, BLDP benign lung disease patients, AC Adenocarcinoma, SC Squamous carcinoma, ASC adenosquamous carcinoma, CTCs circulating tumor cells, CTECs circulating tumor-derived endothelial cells, M median, P25-P75 inter-quartile range

Supplemental Table 2. Statistical parameters of aneuploid CTCs and CTECs of 104 participating subjects

|  | **Aneuploid CTCs (Units/6ml)** | | | | | | | | | | |  | **Aneuploid CTECs (Units/6ml)** | | | | | | | | | | |
| --- | --- | --- | --- | --- | --- | --- | --- | --- | --- | --- | --- | --- | --- | --- | --- | --- | --- | --- | --- | --- | --- | --- | --- |
| **Characteristics** | **Triploid** | | |  | **Tetraploid** | | |  | **Multiploid** | | |  | **Triploid** | | |  | **Tetraploid** | | |  | **Multiploid** | | |
|  | **M** | **P25-P75** | ***P*** |  | **M** | **P25-P75** | ***P*** |  | **M** | **P25-P75** | ***P*** |  | **M** | **P25-P75** | ***P*** |  | **M** | **P25-P75** | ***P*** |  | **M** | **P25-P75** | ***P*** |
| Healthy | 1 | 0-2 | <0.001 |  | 0 | 0-1.75 | <0.0001 |  | 3 | 1-6 | 0.058 |  | 0 | 0-0 | <0.0001 |  | 0 | 0-0 | <0.0001 |  | 3 | 0.25-6 | <0.001 |
| BLDP | 2 | 0-5 |  |  | 1 | 0-3 |  |  | 1 | 0-4 |  |  | 1 | 0-3 |  |  | 0 | 0-3 |  |  | 4 | 1-8 |  |
| NSCLC | 8 | 3.5-12 |  |  | 4 | 1.5-6 |  |  | 3 | 1-7.5 |  |  | 3 | 1-6 |  |  | 2 | 1-4 |  |  | 8 | 4-23 |  |
| Pathological type |  |  | 0.887 |  |  |  | 0.657 |  |  |  | 0.428 |  |  |  | 0.069 |  |  |  | 0.083 |  |  |  | 0.127 |
| AC | 7.5 | 4-13.5 |  |  | 4 | 2-5.75 |  |  | 8 | 4-22 |  |  | 2.5 | 1-6 |  |  | 2 | 0-4 |  |  | 8.5 | 4.25-22 |  |
| SC | 8 | 4-8.75 |  |  | 2 | 0.25-12 |  |  | 10.5 | 5.75-30 |  |  | 4 | 3-5.75 |  |  | 2.5 | 1.25-4 |  |  | 10.5 | 5.75-30 |  |
| ASC | 11 | 1-40 |  |  | 6 | 1-9 |  |  | 12 | 8-106 |  |  | 8 | 8-8 |  |  | 8 | 6-11 |  |  | 12 | 8-106 |  |
| Other | 6.0 | 1-11.0 |  |  | 2.5 | 1-4 |  |  | 1 | 1-1 |  |  | 1.5 | 0-3 |  |  | 1.5 | 1-2 |  |  | 3.5 | 1-6 |  |
| TNM stage |  |  | 0.240 |  |  |  | 0.897 |  |  |  | 0.365 |  |  |  | 0.134 |  |  |  | 0.653 |  |  |  | 0.389 |
| Ⅰ | 9 | 4-14 |  |  | 4 | 2-6 |  |  | 2 | 0-7 |  |  | 2 | 0-6 |  |  | 2 | 1-4 |  |  | 7 | 4-24 |  |
| Ⅱ | 4.5 | 3-6 |  |  | 12 | 1-23 |  |  | 19.5 | 1-38 |  |  | 2 | 1-3 |  |  | 2 | 0-4 |  |  | 7.5 | 3-12 |  |
| Ⅲ | 4 | 2-8 |  |  | 4 | 0-5.5 |  |  | 3 | 2-7.5 |  |  | 5 | 1-6 |  |  | 1 | 0-4 |  |  | 8 | 3-16 |  |
| Ⅳ | 11 | 8-14 |  |  | 3 | 2-6 |  |  | 4 | 2-17 |  |  | 5 | 3-8 |  |  | 3 | 0-7 |  |  | 14 | 8-58 |  |

The number of CTEC triploid in adenosquamous carcinoma was significantly higher than that in adenocarcinoma and squamous carcinoma (*p*=0.0161*, Mann-Whitney U test; *p*=0.0424*, Mann-Whitney U test). The number of CTEC tetraploid in adenosquamous carcinoma was significantly higher than that in adenocarcinoma and squamous carcinoma (*p*=0.0061**, Mann-Whitney U test; *p*=0.0061**, Mann-Whitney U test). The number of CTEC triploid in stage IV was significantly higher than that in stage I (p=0.0276*, Mann-Whitney U test). Measurement data were compared between multiple groups by Kruskal-Wallis test.

Abbreviations: NSCLC non-small cell lung cancer, BLDP benign lung disease patients, AC Adenocarcinoma, SC Squamous carcinoma, ASC adenosquamous carcinoma, CTCs circulating tumor cells, CTECs circulating tumor-derived endothelial cells, M median, P25-P75 inter-quartile range.

Supplemental Table3. The diagnostic sensitivity of CTCs and CTECs in different pathological types and stages of NSCLC

|  | **CTCs** | | | | | | | | | | |  | **CTECs** | | | | | | | | | | |
| --- | --- | --- | --- | --- | --- | --- | --- | --- | --- | --- | --- | --- | --- | --- | --- | --- | --- | --- | --- | --- | --- | --- | --- |
| **Characteristics** | **Total CTCs** | | |  | **_L_CTCs** | | |  | **sCTCs** | | |  | **Total CTECs** | | |  | **_L_CTECs** | | |  | **sCTECs** | | |
|  | **≥11.5** | **＜11.5** | **SEN%** |  | **≥6.5** | **＜6.5** | **SEN%** |  | **≥6.5** | **＜6.5** | **SEN%** |  | **≥10.5** | **＜10.5** | **SEN%** |  | **≥8.5** | **＜8.5** | **SEN%** |  | **≥2.5** | **＜2.5** | **SEN%** |
| Pathological type | |  |  |  |  |  |  |  |  |  |  |  |  |  |  |  |  |  |  |  |  |  |  |
| AC | 27 | 9 | 75.0 |  | 12 | 24 | 33.3 |  | 23 | 13 | 63.9 |  | 27 | 9 | 75.0 |  | 23 | 13 | 63.9 |  | 22 | 14 | 61.1 |
| SC | 6 | 2 | 75.0 |  | 5 | 3 | 62.5 |  | 6 | 2 | 75.0 |  | 7 | 1 | 87.5 |  | 6 | 2 | 75.0 |  | 7 | 1 | 87.5 |
| ASC | 2 | 1 | 66.7 |  | 2 | 1 | 66.7 |  | 2 | 1 | 66.7 |  | 3 | 0 | 100.0 |  | 3 | 0 | 100.0 |  | 3 | 0 | 100.0 |
| Other | 1 | 1 | 50.0 |  | 0 | 2 | 0.0 |  | 1 | 1 | 50.0 |  | 1 | 1 | 50.0 |  | 1 | 1 | 50.0 |  | 1 | 1 | 50.0 |
| TNM stage |  |  |  |  |  |  |  |  |  |  |  |  |  |  |  |  |  |  |  |  |  |  |  |
| Ⅰ | 19 | 8 | 70.4 |  | 10 | 17 | 37.0 |  | 16 | 11 | 59.3 |  | 19 | 8 | 70.4 |  | 17 | 10 | 63.0 |  | 18 | 9 | 66.7 |
| Ⅱ | 1 | 1 | 50.0 |  | 1 | 1 | 50.0 |  | 1 | 1 | 50.0 |  | 2 | 0 | 100.0 |  | 2 | 0 | 100.0 |  | 2 | 0 | 100.0 |
| Ⅲ | 7 | 2 | 77.8 |  | 4 | 5 | 44.4 |  | 6 | 3 | 66.7 |  | 7 | 2 | 77.8 |  | 5 | 4 | 55.6 |  | 5 | 4 | 55.6 |
| Ⅳ | 9 | 2 | 81.8 |  | 4 | 7 | 36.4 |  | 9 | 2 | 81.8 |  | 10 | 1 | 90.9 |  | 9 | 2 | 81.8 |  | 8 | 3 | 72.7 |

Abbreviations: NSCLC non-small cell lung cancer, AC Adenocarcinoma, SC Squamous carcinoma, ASC adenosquamous carcinoma, CTCs circulating tumor cells, CTECs circulating tumor-derived endothelial cells.

Supplemental Table 4. The diagnostic sensitivity of aneuploid CTCs and CTECs in different pathological types and stages of NSCLC

|  | **Aneuploid CTCs** | | | | | | | | | | |  | **Aneuploid CTECs** | | | | | | | | | | |
| --- | --- | --- | --- | --- | --- | --- | --- | --- | --- | --- | --- | --- | --- | --- | --- | --- | --- | --- | --- | --- | --- | --- | --- |
| **Characteristics** | **Triploid** | | |  | **Tetraploid** | | |  | **Multiploid** | | |  | **Triploid** | | |  | **Tetraploid** | | |  | **Multiploid** | | |
|  | **≥6.5** | **＜6.5** | **SEN%** |  | **≥4.5** | **＜4.5** | **SEN%** |  | **≥6.5** | **＜6.5** | **SEN%** |  | **≥3.5** | **＜3.5** | **SEN%** |  | **≥2.5** | **＜2.5** | **SEN%** |  | **≥7.5** | **＜7.5** | **SEN%** |
| Pathological type | |  |  |  |  |  |  |  |  |  |  |  |  |  |  |  |  |  |  |  |  |  |  |
| AC | 19 | 17 | 52.8 |  | 12 | 14 | 33.3 |  | 9 | 27 | 33.3 |  | 16 | 20 | 44.4 |  | 15 | 11 | 41.7 |  | 19 | 14 | 52.8 |
| SC | 6 | 2 | 75.0 |  | 2 | 6 | 25.0 |  | 3 | 5 | 37.5 |  | 4 | 4 | 50.0 |  | 4 | 4 | 50.0 |  | 6 | 2 | 75.0 |
| ASC | 2 | 1 | 66.7 |  | 2 | 1 | 66.7 |  | 2 | 1 | 66.7 |  | 3 | 0 | 100.0 |  | 3 | 0 | 100.0 |  | 3 | 0 | 100.0 |
| Other | 1 | 1 | 50.0 |  | 1 | 1 | 50.0 |  | 0 | 2 | 0.0 |  | 0 | 2 | 0.0 |  | 0 | 2 | 0.0 |  | 0 | 1 | 0.0 |
| TNM stage |  |  |  |  |  |  |  |  |  |  |  |  |  |  |  |  |  |  |  |  |  |  |  |
| Ⅰ | 16 | 9 | 59.3 |  | 10 | 17 | 37.0 |  | 8 | 19 | 29.6 |  | 11 | 16 | 40.7 |  | 11 | 16 | 40.7 |  | 13 | 14 | 48.1 |
| Ⅱ | 0 | 2 | 0.0 |  | 1 | 1 | 50.0 |  | 1 | 1 | 50.0 |  | 0 | 2 | 0.0 |  | 1 | 1 | 50.0 |  | 1 | 1 | 50.0 |
| Ⅲ | 3 | 6 | 33.3 |  | 2 | 7 | 22.2 |  | 2 | 7 | 22.2 |  | 6 | 3 | 66.7 |  | 3 | 6 | 33.3 |  | 5 | 4 | 55.6 |
| Ⅳ | 9 | 2 | 81.8 |  | 3 | 8 | 27.3 |  | 3 | 8 | 27.3 |  | 7 | 4 | 63.6 |  | 7 | 4 | 63.6 |  | 9 | 2 | 81.8 |

Abbreviations: NSCLC non-small cell lung cancer, AC Adenocarcinoma, SC Squamous carcinoma, ASC adenosquamous carcinoma, CTCs circulating tumor cells, CTECs circulating tumor-derived endothelial cells.

**Supplemental table5. Target gene information of ctDNA panel**

| *ABL1* | *AKT1* | *AKT2* | *ALK* | *APC* | *AR* | *ARAF* |
| --- | --- | --- | --- | --- | --- | --- |
| *BRAF* | *BRCA1* | *BRCA2* | *CCND1* | *CCND2* | *CCND3* | *CD274* |
| *CDK4* | *CDK6* | *CDKN2A* | *CSF1R* | *CTNNB1* | *DDR2* | *DPYD* |
| *EGFR* | *ERBB2* | *ESR1* | *EZH2* | *FBXW7* | *FGFR1* | *FGFR2* |
| *FGFR3* | *FLT1* | *FLT3* | *FLT4* | *GATA3* | *GNA11* | *GNAQ* |
| *GNAS* | *IDH1* | *IDH2* | *JAK2* | *JAK3* | *KDR* | *KEAP1* |
| *KIT* | *KRAS* | *MAP2K1* | *MAP2K2* | *MET* | *MLH1* | *MSH2* |
| *MSH6* | *MTOR* | *NF2* | *NFE2L2* | *NRAS* | *NTRK1* | *PDCD1LG2* |
| *PDGFRA* | *PDGFRB* | *PIK3CA* | *PIK3R1* | *PMS2* | *PTCH1* | *PTEN* |
| *RAF1* | *RB1* | *RET* | *RNF43* | *ROS1* | *SMAD4* | *SMO* |
| *STK11* | *TERT* | *TP53* | *TSC1* | *TSC2* | *UGT1A1* | *VHL* |

**Supplemental table 6. Target gene information of tDNA panel**

| *ABCB1(MDR1)* | *ABCC2* | *ABL1* | *ADH1B* | *AIP* | *AKT1* | *AKT2* |
| --- | --- | --- | --- | --- | --- | --- |
| *AKT3* | *ALDH2* | *ALK* | *AMER1* | *APC* | *APEX1* | *AR* |
| *ARAF* | *ARID1A* | *ARID1B* | *ARID2* | *ARID5B* | *ASCL4* | *ASXL1* |
| *ATF1* | *ATIC* | *ATM* | *ATR* | *ATRX* | *AURKA* | *AURKB* |
| *AXIN2* | *AXL* | *B2M* | *BAD* | *BAI3* | *BAK1* | *BAP1* |
| *BARD1* | *BAX* | *BCL2* | *BCL2L1* | *BCL2L11* | *BCL3* | *BCR* |
| *BIRC3* | *BLM* | *BMPR1A* | *BRAF* | *BRCA1* | *BRCA2* | *BRD4* |
| *BRIP1* | *BTG2* | *BTK* | *BUB1B* | *CLLOR30* | *CASP8* | *CBL* |
| *CBLB* | *CCND1* | *CCNE1* | *CD274(PD-1)* | *CD74* | *CDA* | *CDC73* |
| *CDH1* | *CDK10* | *CDK12* | *CDK4* | *CDK6* | *CDK8* | *CDKN1A* |
| *CDKN1B* | *CDKN1C* | *CDKN2A* | *CDKN2B* | *CDKN2C* | *CEBPA* | *CEP57* |
| *CHD4* | *CHD8* | *CHEK1* | *CHEK2* | *CREBBP* | *CRKL* | *CSF1R* |
| *CTCF* | *CTLA4* | *CTNNB1* | *CUL3* | *CUX1* | *CXCL8* | *CXCR4* |
| *CYLD* | *CYP19A1* | *CYP2A13* | *CYP2A6* | *CYP2A7* | *CYP2B6*6* | *CYP2C19*2* |
| *CYP2C9*3* | *CYP2D6* | *CYP3A4*4* | *CYP3A5* | *CYSLTR2* | *DAXX* | *DDR2* |
| *DENND1A* | *DHFR* | *DICER1* | *DLL3* | *DNMT3A* | *DOT1L* | *DPYD* |
| *DTL(CDT2)* | *DUSP2* | *EGFR* | *EIF1AX* | *EP300* | *EPAS1* | *EPCAM* |
| *EPHA2* | *EPHA3* | *EPHA5* | *ERBB2* | *ERBB2IP* | *ERBB3* | *ERBB4* |
| *ERCC1* | *ERCC2* | *ERCC3* | *ERCC4* | *ERCC5* | *ESR1* | *ETV1* |
| *ETV4* | *ETV5* | *ETV6* | *EWSR1* | *EXT1* | *EXT2* | *EZH2* |
| *EZR* | *FANCA* | *FANCC* | *FANCD2* | *FANCE* | *FANCF* | *FANCG* |
| *FANCI* | *FANCL* | *FANCM* | *FAT1* | *FBXW7* | *FGF19* | *FGFR1* |
| *FGFR2* | *FGFR3* | *FGFR4* | *FH* | *FLCN* | *FLT1* | *FLT3* |
| *FLT4* | *FOXA1* | *FOXL2* | *FOXO3* | *FOXP1* | *FRG1* | *GATA1* |
| *GATA2* | *GATA3* | *GATA4* | *GATA6* | *GNA11* | *GNAQ* | *GNAS* |
| *GR1N2A* | *GRM3* | *GRM8* | *GSTM1* | *GSTM4* | *GSTP1* | *GSTT1* |
| *HDAC1* | *HDAC2* | *HDAC9* | *HGF* | *HLA-A* | *HMOX1* | *HNF1A* |
| *HNF1B* | *HRAS* | *HSPB1* | *IDH1* | *IDH2* | *IFNA6* | *IFNB1* |
| *IFNE* | *IFNG* | *IFNGR1* | *IFNGR2* | *IGF1R* | *IGF2* | *IKBKE* |
| *IKZF1* | *IL13* | *IL1A* | *IL7R* | *INPP4B* | *IRF1* | *IRF2* |
| *ITGB6* | *JAK1* | *JAK2* | *JAK3* | *JARID2* | *JUN* | *KDM5A* |
| *KDR(VEFGR2)* | *KEAP1* | *KIF1B* | *KIT* | *KITLG* | *KLLN* | *KMT2A* |
| *KMT2B* | *KMT2C* | *KMT2D* | *KRAS* | *LHCGR* | *LIG3* | *LIG4* |
| *LIN28B* | *LMO1* | *LRP1B* | *LYN* | *LZTR1* | *MALT1* | *MAP2K1* |
| *MAP2K2* | *MAP2K4* | *MAP3K1* | *MAP3K4* | *MAPK1* | *MAPK3* | *MAX* |
| *MCL1* | *MDM2* | *MDM4* | *MECOM* | *MED12* | *MEF2B* | *MEN1* |
| *MET* | *MGMT* | *MIF* | *MITF* | *MLH1* | *MLH3* | *MLLT1* |
| *MLLT3* | *MLL4* | *MMP1* | *MPL* | *MRE11A* | *MSH2* | *MSH6* |
| *MTHFR* | *MTOR* | *MTRR* | *MUC5B* | *MUTYH* | *MYC* | *MYCL* |
| *MYCN* | *MYD88* | *MYH9* | *NAT1* | *NBN* | *NCOR1* | *NEIL1* |
| *NF1* | *NF2* | *NFE2L2* | *NFKB1* | *NFKBIA* | *NKX2-1* | *NOS2* |
| *NOS3* | *NOTCH1* | *NOTCH2* | *NOTCH3* | *NPM1* | *NQO1* | *NRAS* |
| *NRG1* | *NSD1* | *NTHL1* | *NTRK1* | *NTRK2* | *NTRK3* | *NUTM1* |
| *PAK2* | *PAK3* | *PALB2* | *PALLD* | *PARK2* | *PARP1* | *PARP2* |
| *PAX5* | *PBRM1* | *PDCD1* | *PDCD1LG2* | *PDE11A* | *PDGFRA* | *PDGFRB* |
| *PDK1* | *PGR* | *PHOX2B* | *PIK3C3* | *PIK3CA* | *PIK3CD* | *PIK3R1* |
| *PIK3R2* | *PKHD1* | *PLAG1* | *PLCB4* | *PLK1* | *PMAIP1* | *PMS1* |
| *PMS2* | *PNKP(PNK)* | *POLD1* | *POLD3* | *POLE* | *POLH* | *POLT1* |
| *PPARD* | *PPP2R1A* | *PRDM1* | *PREX2* | *PRF1* | *PRKACA* | *PRKAR1A* |
| *PRKCB* | *PRKCI* | *PRKDC* | *PRSS1* | *PRSS3* | *PTCH1* | *PTEN* |
| *PTK2* | *PTPN11* | *PTPN13* | *QKI* | *RAC1* | *RAC3* | *RAD50* |
| *RAD51* | *RAD51B* | *RAD51C* | *RAD51D* | *RAD54L* | *RAD9A* | *RAF1* |
| *RARA* | *RARG* | *RASGEF1A* | *RB1* | *RCC1* | *RECQL4* | *RELA* |
| *RELN* | *RET* | *RHOA* | *RICTOR* | *RNF43* | *ROS1* | *RPTOR* |
| *RRM1* | *RUNX1* | *RUNX1T1* | *SBDS* | *SDC4* | *SDHA* | *SDHB* |
| *SDHC* | *SDHD* | *SEPT9* | *SERPINE1* | *SETBP1* | *SETD2* | *SF3B1* |
| *SGK1* | *SKP2* | *SLC34A2* | *SLC3A2* | *SMAD2* | *SMAD3* | *SMAD4* |
| *SMAD7* | *SMARCA4* | *SMARCB1* | *SMO* | *SOCS1* | *SOS1* | *SOX2* |
| *SPOP* | *SPRED1* | *SPRY4* | *SRC* | *SRSF2* | *SRY* | *STAG2* |
| *STAT1* | *STAT3* | *STK11* | *STMN1* | *SUFU* | *SUMO1* | *TACC3* |
| *TAP1* | *TAP2* | *TBK1* | *TEK* | *TEKT4* | *TERC* | *TERT* |
| *TET2* | *TFG* | *TGFB1* | *TGFBR2* | *THADA* | *TMEM127* | *TMEM167A* |
| *TMPRSS2* | *TNF* | *TNFAIP3* | *TNFRSF11A* | *TNFRSF14* | *TNFRSF19* | *TNFRSF1B* |
| *TNFSF11* | *TOP1* | *TOP2A* | *TP53* | *TP63* | *TPMT* | *TSC1* |
| *TSC2* | *TSHR* | *TTF1* | *TUBB3* | *TYMS* | *USAF1* | *UGT1A1* |
| *UNG* | *VAMP2* | *VEGFA* | *VHL* | *WAS* | *WISP3* | *WRN* |
| *WT1* | *XPA* | *XPC* | *XRCC1* | *XRCC2* | *XRCC3* | *XRCC4* |
| *XRCC5* | *YAP1* | *ZNF2* | *ZNF217* | *ZNF703* | *-* | *-* |

| Gene | All Subjects  n (%)(N=28) | Pathological  type | Variant  CDSchange | Variant  type | Variant  Description | Alle  Fraction(%) |
| --- | --- | --- | --- | --- | --- | --- |
| ***TP53*** | 9(32.1) | AC | exon4: c.374C>A: p.Thr125Lys | SNV | Missense | 0.38 |
|  |  | AC | Intron7: c.919+1G>A: …… | SNV | Intronic | 0.29 |
|  |  | SCC | exon6: c.646G>T: p.Val216Leu | SNV | Missense | 0.12 |
|  |  | SCC | exon7: c.701A>G: p.Tyr234Cys | SNV | Missense | 7.29 |
|  |  | SCC | exon6: c.592G>T: p.Glu198* | SNV | Nonsense | 13.85 |
|  |  | SCC | exon6: c.583A>T: p.Ile195Phe | SNV | Missense | 29.97 |
|  |  | SCC# | exon6: c.578A>G: p.His193Arg | SNV | Missense | 7.23 |
|  |  | SCC# | exon7: c.711G>A: p.Met237Ile | SNV | Missense | 3.88 |
|  |  | SCC | exon6: c.583A>T: p.Ile195Phe | SNV | Missense | 0.52 |
|  |  | SCC | exon7: c.713G>A: p.Cys238Tyr | SNV | Missense | 0.16 |
| ***RB1*** | 2(7.1) | Benign | exon19: c.1862G>A: p.Arg621His | SNV | Missense | 48.9 |
|  |  | SCC | Intron20: c.2107-1G>A: …… | SNV | Intronic | 13.3 |
| ***ERBB2*** | 2(7.1) | Benign | exon23: c.2810A>G: p.Lys937Arg | SNV | Missense | 0.34 |
|  |  | SCC | exon23: c.2810A>G: p.Lys937Arg | SNV | Missense | 0.37 |
| ***IDH1*** | 2(7.1) | Benign | exon4: c.394C>A: p.Arg132Ser | SNV | Missense | 0.05 |
|  |  | AC | exon4: c.394C>G: p.Arg132Gly | SNV | Missense | 2.16 |
| ***PTEN*** | 2(7.1) | Benign | exon8: c.962_963insA: p.Asn323fs | Delins | Frameshift | 0.12 |
|  |  | SCC | Intron8: c.1026+1G>C: …… | SNV | Intronic | 1.77 |
| ***MET*** | 2(7.1) | Benign | exon14: c.3013C>G: p.Arg1005Gly | SNV | Missense | 0.09 |
|  |  | SCC | CNV SCORE:4.75 | CNV | - | - |
| ***ALK*** | 2(7.1) | AC | exon12: c.2073C>T: p.Ser691Ser | SNV | Synonymous | 42.63 |
|  |  | SCC | exon28: c.4076A>G: p.Tyr1359Cys | SNV | Missense | 6.85 |
| ***FGFR2*** | 2(7.1) | SCC | exon12: c.1650T>A: p.Asn549Lys | SNV | Missense | 0.52 |
|  |  | SCC | exon9: c.1147T>C: p.Cys383Arg | SNV | Missense | 0.08 |
| ***RET*** | 1(3.6) | AC | exon11: c.1891G>A: p.Asp631Asn | SNV | Missense | 41.54 |
| ***PTCH1*** | 1(3.6) | AC | Intron9: c.1347+6G>A: …… | SNV | Intronic | 45.65 |
| ***DDR2*** | 1(3.6) | AC | exon14: c.1530C>T: p.Val510Val | SNV | Synonymous | 45.07 |
| ***TSC2*** | 1(3.6) | SCC | exon20: c.2153G>A: p.Arg718His | SNV | Missense | 44.64 |
| ***MSH6*** | 1(3.6) | SCC | exon5: c.3197A>G: p.Tyr1066Cys | SNV | Missense | 0.07 |
| ***GNAS*** | 1(3.6) | SCC | exon8: c.604C>T: p.Arg201Cys | SNV | Missense | 0.92 |
| ***NFE2L2*** | 1(3.6) | SCC | exon2: c.92G>C: p.Gly31Ala | SNV | Missense | 15.43 |
| ***KEAP1*** | 1(3.6) | SCC | exon4: c.1408C>T: p.Arg470Cys | SNV | Missense | 15.15 |
| ***EGFR*** | 1(3.6) | SCC | CNV SCORE:3.79 | CNV | - | - |
| ***MAP2K1*** | 1(3.6) | SCC | exon6: c.644T>C: p.Leu215Pro | SNV | Missense | 0.06 |
| ***PIK3CA*** | 1(3.6) | SCC | exon21: c.3140A>G: p.His1047Arg | SNV | Missense | 0.11 |
| ***FGFR3-TACC3*** | 1(3.6) | SCC | Fusion | - | - | - |

**Supplemental Table7. Mutational profile of ctDNA in enrolled 28 patients**

The genes name in bold means in the list of 75 overlapping genes between ctDNA and tDNA panel,

# Two different mutations in the same patient

**Supplemental Table8. Mutational profiles of tumor DNA in filtered 9 patients**

| Gene | All Subjects  n (%) (N=9) | Pathological  type | Variant  CDSchange | Variant  type | Variant  Description | Alle  Fraction(%) |
| --- | --- | --- | --- | --- | --- | --- |
| ***EGFR*** | 5(55.6) | AC | exon21:c.2573T>G: p.L858R | SNV | Missense | 55.2 |
|  |  | AC | exon21:c.2573T>G: p.L858R | SNV | Missense | 15.3 |
|  |  | AC | exon21:c.2573T>G: p.L858R | SNV | Missense | 23.1 |
|  |  | AC | exon21:c.2573T>G: p.L858R | SNV | Missense | 21.2 |
|  |  | SCC | exon19:c.2237_2255delinsT:p.E746_S752delinsV | Delins | nonFrameshift | 39.5 |
| *MCL1* | 4(44.4) | AC | 2.6 copies | CNV | - | - |
|  |  | AC | 2.6 copies | CNV | - | - |
|  |  | AC | 6.4 copies | CNV | - | - |
|  |  | AC | 2.4 copies | CNV | - | - |
| ***CDK4*** | 3(33.3) | AC | 7.6 copies | CNV | - | - |
|  |  | AC | 2.1 copies | CNV | - | - |
|  |  | AC | 2.3 copies | CNV | - | - |
| *VEGFA* | 2(22.2) | AC | 7.4 copies | CNV | - | - |
|  |  | AC | 2.2 copies | CNV | - | - |
| *BCL3* | 2(22.2) | AC | 9.8 copies | CNV | - | - |
|  |  | AC | 2.3 copies | CNV | - | - |
| ***FGFR1*** | 2(22.2) | AC | exon13:c.1727G>A: p.R576Q | SNV | Missense | 63.0 |
|  |  | SCC | exon5:c.565C>T:p.R189C | SNV | Missense | 9.9 |
| *MYC* | 2(22.2) | AC | 2.2 copies | CNV | - | - |
|  |  | SCC | 2.1 copies | CNV | - | - |
| ***PDGFRB*** | 2(22.2) | AC | exon7: c.1084G>A: p.A362T | SNV | Missense | 1.3 |
|  |  | SCC | exon15: c.2129C>T:p.P710L | SNV | Missense | 8.8 |
| *CYLD* | 2(22.2) | SCC | exon4:c.173C>G:p.S58* | SNV | Nonsense | 34.1 |
|  |  | SCC | exon10:c.1145A>T:p.E382V | SNV | Missense | 2.6 |
| ***TP53*** | 2(22.2) | SCC | exon6:c.646G>T:p.V216L | SNV | Missense | 36.5 |
|  |  | SCC | exon8:c.833C>T:p.P278L | SNV | Missense | 39.0 |
| ***FGFR2*** | 1(11.1) | SCC | exon3:c.290C>T:p.A97V | SNV | Missense | 9.0 |
| *DUSP2* | 1(11.1) | Benign | exon4: c.914T>A: p.L305Q | SNV | Missense | 46.4 |
| *CDA* | 1(11.1) | Benign | exon2: c.160A>T: p.N54Y | SNV | Missense | 47.2 |
| ***RB1*** | 1(11.1) | Benign | exon19: c.1862G>A:p.R621H | SNV | Missense | 49.8 |
| ***RET*** | 1(11.1) | AC | exon11: c.1891G>A:p.D631N | SNV | Missense | 41.9 |
| *ARID1A* | 1(11.1) | AC | exon18:c.4798_4799delTCinsAA: p.S1600N | Delins | Missense | 13.1 |
| *CUL3* | 1(11.1) | AC | exon9: c.1288C>T: p.R430C | SNV | Missense | 35.4 |
| *MLH1* | 1(11.1) | AC | exon12:c.1404C>A: p.N468K | SNV | Missense | 62.2 |
| *STMN1* | 1(11.1) | AC | 2.7 copies | CNV | - | - |
| *MDM4* | 1(11.1) | AC | 2.5 copies | CNV | - | - |
| *CBL* | 1(11.1) | AC | exon9: c.1384C>T: p.R462* | SNV | Nonsense | 2.1 |
| *POLD1* | 1(11.1) | AC | Single copy | CNV | - | - |
| *GATA6* | 1(11.1) | AC | exon2: c.145G>A: p.G49S | SNV | Missense | 35.6 |
| *NSD1* | 1(11.1) | AC | exon23: c.7493G>A: p.G2498E | SNV | Missense | 22.1 |
| *RAD50* | 1(11.1) | AC | exon21: c.3278G>T: p.R1093L | SNV | Missense | 21.2 |
| *MDM2* | 1(11.1) | AC | 2.8 copies | CNV | - | - |
| *TOP1* | 1(11.1) | AC | exon15:c.1517C>G: p.S506* | SNV | Nonsense | 2.4 |
| ***MLH1*** | 1(11.1) | AC | exon12:c.1404C>A: p.N468K | SNV | Missense | 36.2 |
| *BMPR1A* | 1(11.1) | SCC | Single copy | CNV | - | - |
| *PALLD* | 1(11.1) | SCC | exon10: c.1847_1859delGCCGTG  GAGTAAA: p.S616Mfs*3 | Delins | Frameshift | 32.0 |
| *PRKAR1A* | 1(11.1) | SCC | exon2:c.46C>T:p.R16* | SNV | Nonsense | 15.9 |
| ***PTEN*** | 1(11.1) | SCC | exon9:c.1030A>T:p.K344* | SNV | Nonsense | 34.2 |
| *WISP3* | 1(11.1) | SCC | exon2:c.220C>T:p.Q74* | SNV | Nonsense | 3.3 |
| *ATR* | 1(11.1) | SCC | exon14:c.2963A>G:p.N988S | SNV | Missense | 20.8 |
| *CBLB* | 1(11.1) | SCC | exon5:c.607G>A:p.K7E203K | SNV | Missense | 2.1 |
| *DOT1L* | 1(11.1) | SCC | exon20:c.2327C>G:p.P776R | SNV | Missense | 31.4 |
| *FANCC* | 1(11.1) | SCC | exon5:c.406C>G:p.Q136E | SNV | Missense | 2 |
| *GATA2* | 1(11.1) | SCC | exon2:c.206G>T:p.R69L | SNV | Missense | 21.3 |
| *IFNGR1* | 1(11.1) | SCC | exon7:c.1187C>G:p.S396W | SNV | Missense | 4.0 |
| *MED12* | 1(11.1) | SCC | exon15:c.2177A>C:p.Y726S | SNV | Missense | 2.0 |
| *NPM1* | 1(11.1) | SCC | exon10:c.844G>A:p.E282K | SNV | Missense | 34.0 |
| ***ROS1*** | 1(11.1) | SCC | exon4:c.265_266delGGinsTT:p.G89F | Delins | Missense | 40.3 |
| *WRN* | 1(11.1) | SCC | exon26:c.3194C>T:p.A1065V | SNV | Missense | 3.1 |
| *HARS* | 1(11.1) | SCC | exon2:c.35G>A:p.G12D | SNV | Missense | 17.3 |
| *LRP1B* | 1(11.1) | SCC | exon78:c.11952C>G:p.Y3984* | SNV | Nonsense | 27.2 |
| *ARID1B* | 1(11.1) | SCC | exon12:c.3185A>G:p.E1062G | SNV | Missense | 31.5 |
| *AXIN2* | 1(11.1) | SCC | exon2:c.325G>A:p.D109N | SNV | Missense | 10.2 |
| *BRIP1* | 1(11.1) | SCC | exon17:c.2387T>C:p.L796P | SNV | Missense | 37.8 |
| *EPHA3* | 1(11.1) | SCC | exon14:c.2362A>T:p.I788F | SNV | Missense | 37.7 |
| *FANCL* | 1(11.1) | SCC | exon14:c.1124A>C:p.H375P | SNV | Missense | 2.7 |
| ***FLT3*** | 1(11.1) | SCC | exon12:c.1435A>G:p.T479A | SNV | Missense | 35.6 |
| ***FLT4*** | 1(11.1) | SCC | exon15:c.2228C>T:p.A743V | SNV | Missense | 2.0 |
| ***TSC2*** | 1(11.1) | SCC | exon20: c.2153G>A:p.R718H | SNV | Missense | 47.8 |
| *LYN* | 1(11.1) | SCC | exon13:c.1372C>T:p.Q458* | SNV | Nonsense | 27.7 |
| *MYCN* | 1(11.1) | SCC | exon3:c.1172G>T:p.R391L | SNV | Missense | 22.4 |
| *PAK3* | 1(11.1) | SCC | exon14:c.1274C>A:p.T425N | SNV | Missense | 10.7 |
| *EP300* | 1(11.1) | SCC | exon24:c.3939_3940delGA:p.N1314Sfs*3 | Delins | Frameshift | 11.1 |
| ***GNAS*** | 1(11.1) | SCC | 2.1 copies | CNV | - | - |
| *QKI* | 1(11.1) | SCC | exon3:c.401delA:p.K134Rfs*14 | Delins | Frameshift | 8.4 |
| *SMARCA4* | 1(11.1) | SCC | exon19:c.2729C>T:p.T910M | SNV | Missense | 9.5 |
| *SPRY4* | 1(11.1) | SCC# | exon3:c.579_580delTG:p.A194Ifs*42 | Delins | Frameshift | 10.3 |
|  |  | SCC# | exon3:c.113C>A:p.S38* | SNV | Nonsense | 1.0 |
| ***ABL1*** | 1(11.1) | SCC# | exon10:c.1651C>T:p.P551S | SNV | Missense | 4.2 |
|  |  | SCC# | exon11:c.2770G>A:p.G924R | SNV | Missense | 1.0 |
| ***AKT2*** | 1(11.1) | SCC | exon10:c.839A>C:p.N280T | SNV | Missense | 2.6 |
| ***CSF1R*** | 1(11.1) | SCC | exon4:c.363G>T:p.Q121H | SNV | Missense | 9.0 |
| *ETV4* | 1(11.1) | SCC | exon7:c.455A>G:p.Q152R | SNV | Missense | 8.0 |
| *GRM3* | 1(11.1) | SCC | exon4:c.2166G>T:p.K722N | SNV | Missense | 1.5 |
| *LZTR1* | 1(11.1) | SCC | exon18:c.2173C>T:p.R725C | SNV | Missense | 2.8 |
| *POLE* | 1(11.1) | SCC | exon8:c.743A>G:p.Y248C | SNV | Missense | 8.3 |
| *SEPT9* | 1(11.1) | SCC | exon2:c.547G>A:p.A183T | SNV | Missense | 1.2 |
| *THADA* | 1(11.1) | SCC | exon33:c.4868C>T:p.T1623M | SNV | Missense | 1.4 |
| *XRCC2* | 1(11.1) | SCC | exon3:c.404G>A:p.S135N | SNV | Missense | 33.1 |
| *YAP1* | 1(11.1) | SCC | exon6:c.1058A>G:p.Y353C | SNV | Missense | 1.6 |
| *CYP2A6* | 1(11.1) | SCC | exon1:c.100C>T:p.P34S | SNV | Missense | 2.1 |
| *PNKP* | 1(11.1) | SCC | exon2:c.53delG:p.G18Efs*21 | Delins | Frameshift | 6.4 |
| *LIG3* | 1(11.1) | SCC | exon3:c.643G>A:p.A215T | SNV | Missense | 7.5 |
| *RELA* | 1(11.1) | SCC | exon10:c.989G>A:p.R330H | SNV | Missense | 1.4 |

The genes name in bold means in the list of 75 overlapping genes between ctDNA and tDNA panel,

# Two different mutations in the same patient

**Supplemental Table 9. Target gene information of HRR panel**

| *AR* | *ATM* | *ATR* | *BARD1* | *BRCA1* | *BRCA2* | *BRIP1* |
| --- | --- | --- | --- | --- | --- | --- |
| *CDH1* | *CDK12* | *CHEK1* | *CHEK2* | *ESR1* | *FANCA* | *FANCL* |
| *HDAC2* | *HOXB13* | *MRE11A* | *NBN* | *PALB2* | *PPP2R2A* | *PTEN* |
| *RAD51B* | *RAD51C* | *RAD51D* | *RAD54L* | *STK11* | *TP53* |  |
| *BRAF* | *ERBB2* | *KRAS* | *NRAS* | *PIK3CA* |  |  |

The genes from *AR* to *TP53* are related to the homologous recombination repair (HRR) pathway, covering the coding region and the exon-intron junction region. The coverage range of extra five important tumor-related driver genes (*BRAF ERBB2 KRAS NRAS* and *PIK3CA)* is as follows: *BRAF*, 11/12/15/18 exon hotspot regions; *ERBB2*, 1/3/8/9/11/16~21/23/25/27 exon hotspot regions; *KRAS&NRAS*, 2/3/4 exon hotspot regions; *PIK3CA*, 2/5/6/8/10/14/21 exon hotspot regions.

**Supplemental Table 10. Target gene information of 61 GENE panel**

| *APC* | *ATM* | *BARD1* | *BMPR1A* | *BRAF* | *BRCA1* | *BRCA2* | *BRIP1* | *CDH1* |
| --- | --- | --- | --- | --- | --- | --- | --- | --- |
| *CDK4* | *CDKN2A* | *CHEK2* | *ELAC2* | *EPCAM* | *FANCC* | *FH* | *FLCN* | *GNAS* |
| *GREM1* | *HOXB13* | *HRAS* | *KIT* | *MAX* | *MEN1* | *MET* | *MLH1* | *MRE11* |
| *MSH2* | *MSH6* | *MUTYH* | *NBN* | *NF1* | *NTRK1* | *PALB2* | *PALLD* | *PDGFRA* |
| *PMS2* | *PRKAR1A* | *PTCH1* | *PTEN* | *RAD50* | *RAD51* | *RAD51C* | *RAD51D* | *RB1* |
| *RET* | *SDHA* | *SDHAF2* | *SDHB* | *SDHC* | *SDHD* | *SMAD4* | *SMARCA4* | *SMARCB1* |
| *STK11* | *TMEM127* | *TP53* | *TSC1* | *TSC2* | *VHL* | *WRN* |  |  |

The entire exons of 61 cancer-related genes in peripheral blood, as well as single nucleotide variants (SNVs) and short fragment insertion/deletion variants (INDELs) within the classic splice sites, were covered.

| Gene | All Subjects  n (%)(N=4) | Pathological  type | Variant  CDSchange | Variant  type | Variant  Description | Alle  Fraction(%) | ACMG  Classification |
| --- | --- | --- | --- | --- | --- | --- | --- |
| **RB1** | 1(25%) | Benign | exon19: c.1862G>A: p.Arg621His | SNV | Missense | 47.53 | Uncertain |
| CHEK2 | 1(25%) | Benign | intron10:c.1095+12G>A:p.? | Intronic | - | 44.26 | Uncertain |
| CDK12 | 1(25%) | Benign | exon14:c.4232C>T:p.(Ala1411Val) | SNV | Missense | 51.22 | Uncertain |

**Supplemental Table 11. Mutational profiles of WBCs DNA in benign 4 patients**

The genes name in bold means in the list of 75 overlapping genes between ctDNA and tDNA panel
